# Supplementary material for: Structure of lasso peptide epimerase MslH reveals metal-dependent acid/base catalytic mechanism
Source: Nat Commun. 2023 Aug 8;14:4752. doi: 10.1038/s41467-023-40232-x (PMC10406935; doi:10.1038/s41467-023-40232-x)
Supplement: Supplementary file 1 — Supplementary Information [file 41467_2023_40232_MOESM1_ESM.pdf]

Supplementary Information

Structure of lasso peptide epimerase MslH reveals metal-dependent acid/base catalytic mechanism

Yu Nakashima[1,4], Atsushi Kawakami[2,4], Yasushi Ogasawara[3], Masatoshi Maeki[3], Manabu Tokeshi[3], Tohru Dairi[3,*], and Hiroyuki Morita[1,*]

[1]*Institute of Natural Medicine, University of Toyama, 2630-Sugitani, 930-0194, Toyama, Japan.*

[2]*Graduate School of Chemical Sciences and Engineering, Hokkaido University, N13-W8, Kita-ku, Sapporo, Hokkaido 060-8628, Japan.*

[3]*Graduate School of Engineering, Hokkaido University, N13-W8, Kita-ku, Sapporo, Hokkaido 060-8628, Japan.*

[4]*These authors contributed equally.*

* dairi@eng.hokudai.ac.jp, hmorita@inm.u-toyama.ac.jp

Table of contents

1. Supplementary figures 2-27
2. Supplementary tables 28-35

1. Supplementary figures

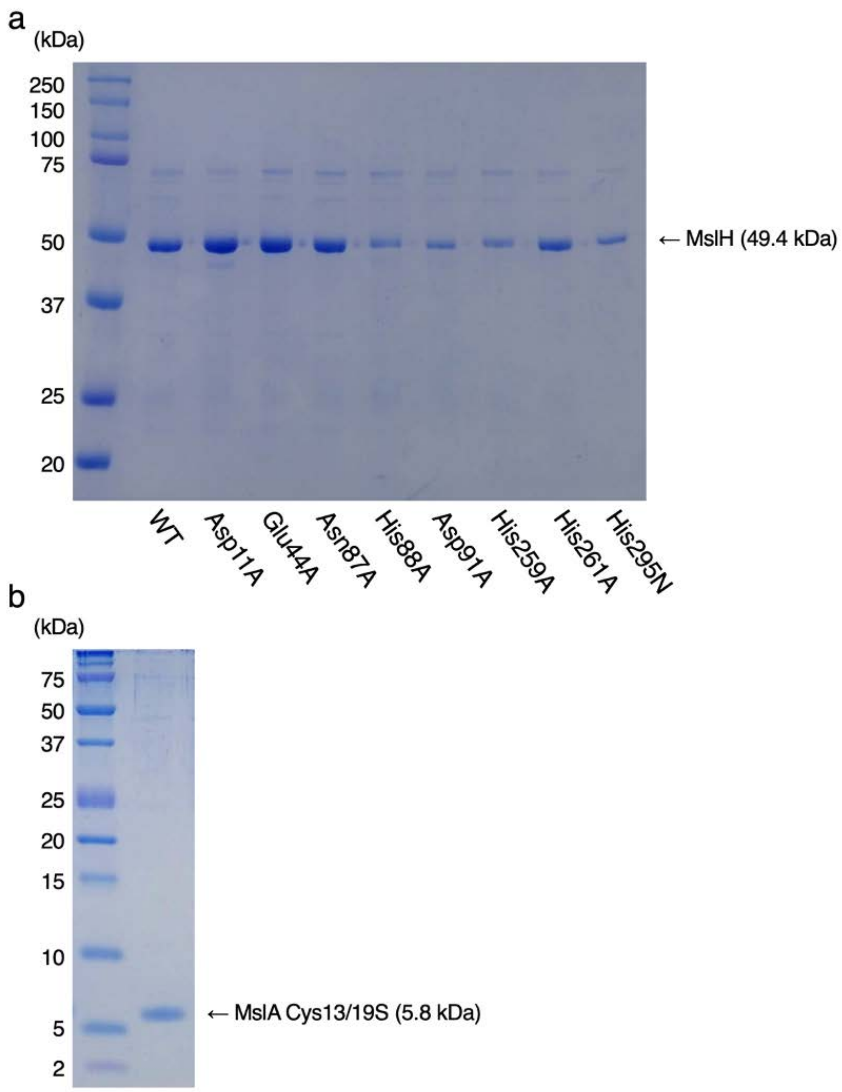

Supplementary Figure 1. SDS-PAGE images for wild-type MslH with variants and the MslA variant. Protein purification levels were monitored based on SDS-PAGE images, using Glycine-SDS-PAGE (10%T, 3%C polyacrylamide gel) for MslH **(a)** and Tricine-SDS-PAGE (16%T, 3%C polyacrylamide gel) for MslA Cys13/19S **(b)**. WT: wild type. At least three independent reactions were performed.

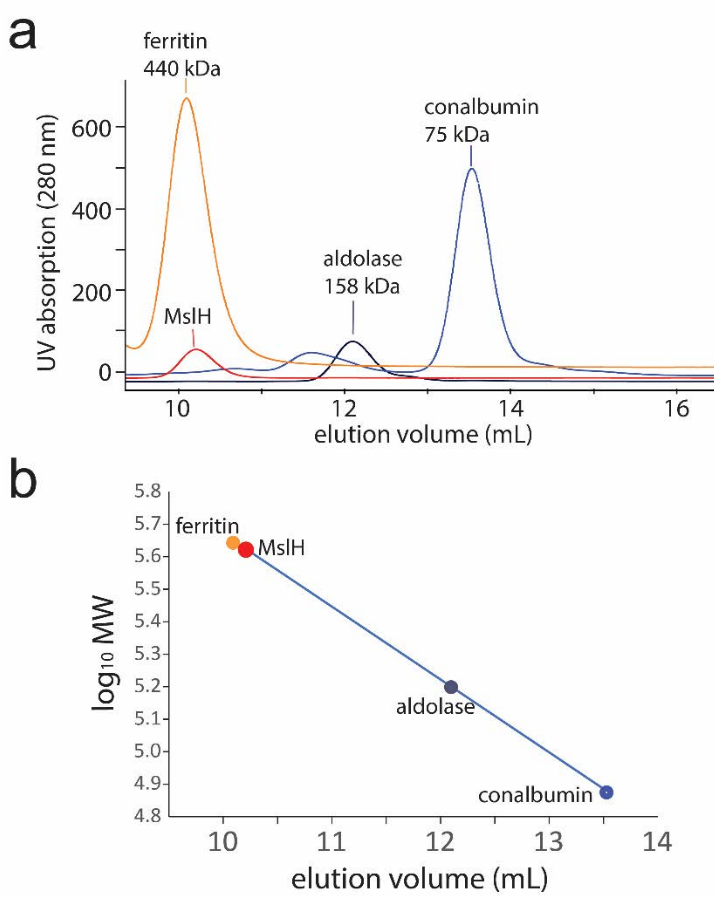

Supplementary Figure 2. Determination of MslH multimer formation by gel-filtration assay. (**a**) UV absorption (280 nm) chromatograms of purified MslH and authentic standard proteins (ferritin: 440 kDa, aldolase: 158 kDa, and conalbumin: 75 kDa in Gel Filtration Calibration Kits HMW (Cytiva) on Superdex 200 Increase 10/300 G column (Cytiva, flow rate: 0.7 mL/min). (**b**) Calibration curve generated with elution volume values (mL) versus log molecular mass values (Da) of standard proteins.

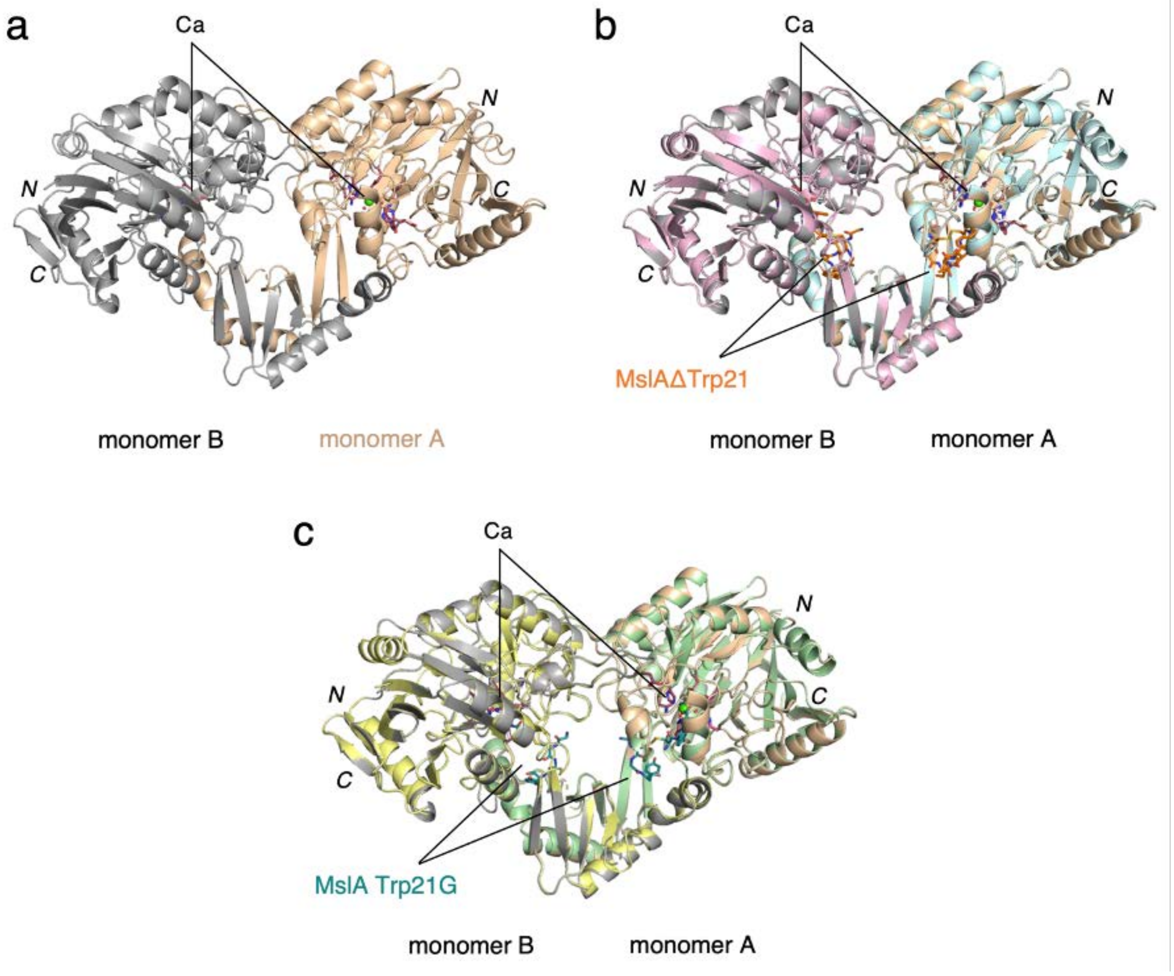

Supplementary Figure 3. Views from the structure of MslH:apo (PDB ID: 8GQ9). Color code: MslH in monomer A: wheat; MslH in monomer B: grey; carbon-backbone of MslH: ruby red; Ca: light green; oxygen: red; nitrogen: blue; w: water molecule.

(**a**) Overview of the dimer formation in the MslH:apo crystal structure. (**b**) Superimposition of the MslH:apo structure with the MslH:MslAΔTrp21 structure (MslH in monomer A: pale cyan; MslH in monomer B: light pink; carbon-backbone of MslAΔTrp21: orange) reveals similar MslH conformations (Cα RMSD = 0.17 Å). (**c**) Superimposition of the MslH:apo structure with the MslH:MslA Trp21G structure (MslH in monomer A: pale green; MslH in monomer B: pale yellow; carbon-backbone of MslA Trp21G: blue green) reveals similar MslH conformations (Cα RMSD = 0.16 Å).

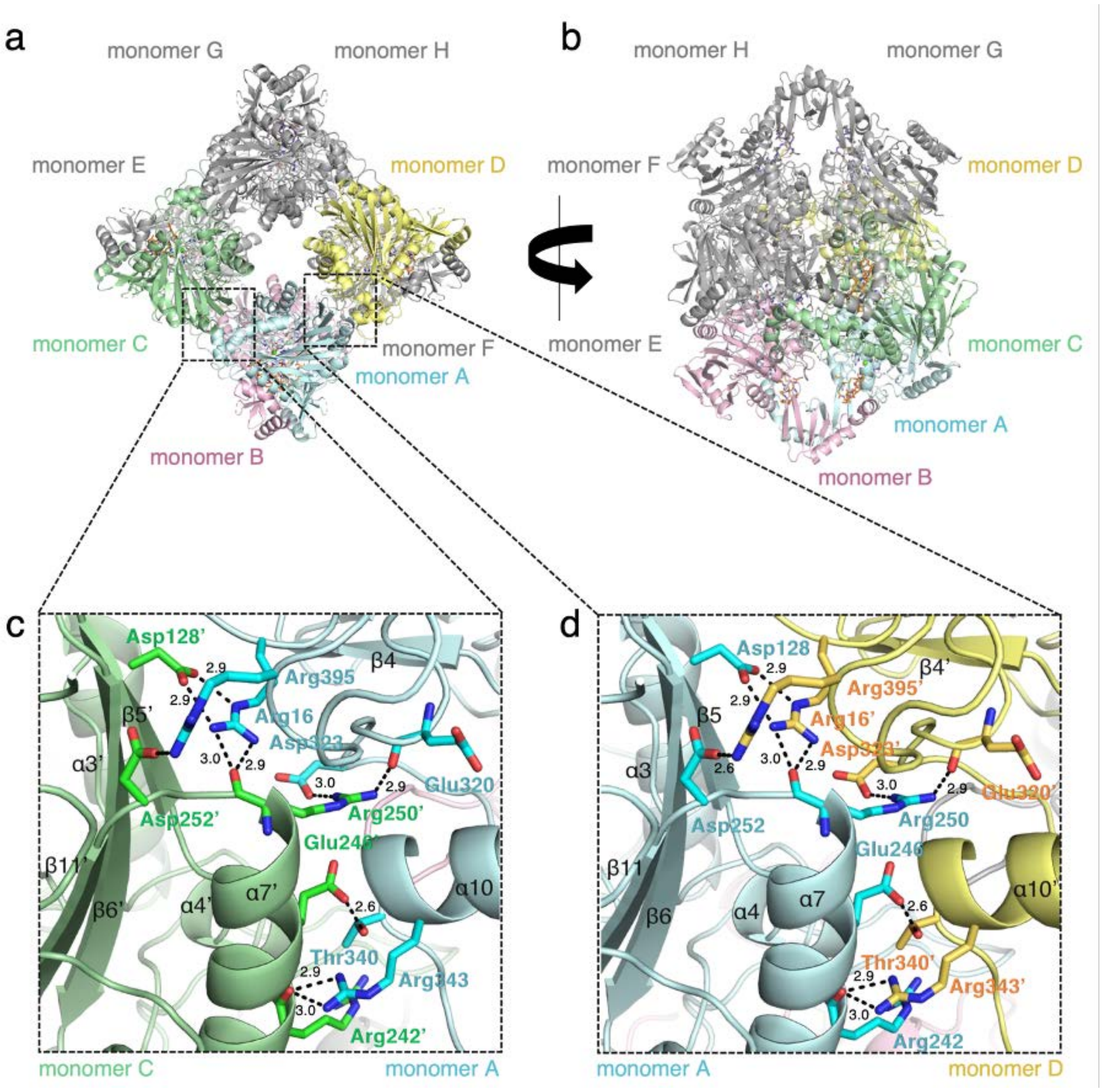

Supplementary Figure 4. Overviews of the octameric MslH structure. Color code: MslH monomer A: pale cyan; MslH monomer B: light pink; MslH monomer C: pale green; MslH monomer D: pale yellow; MslH monomers E-H: grey; oxygen: red; nitrogen: blue.

Overviews of the MslH:MslAΔTrp21 structures as the octamer **(a)** and rotated 90 degrees horizontally **(b)**. Close-up views of the interactions of monomer A with monomer C **(c)** and monomer D **(d)**. Dashed lines represent hydrogen bonds (distances in Å).

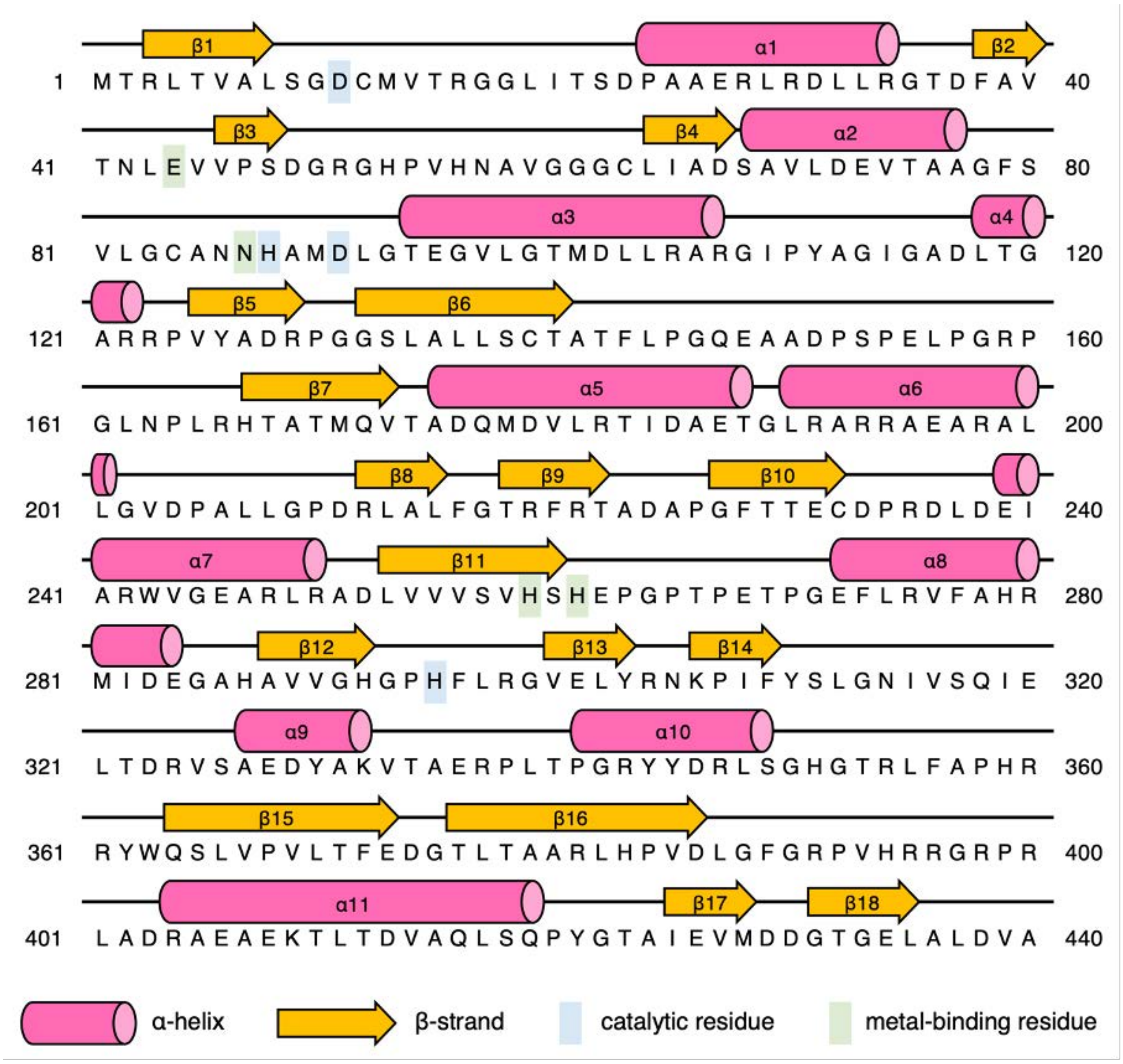

Supplementary Figure 5. The amino acid sequence of MslH. The secondary structure elements of MslH are shown by pink cylinders (α-helices) and yellow arrows (β-sheets). Four catalytic residues, Asp11, His88, Asp91, and His295, involved in epimerization are shown as blue rectangles, and four metal-binding residues, Glu44, Asn87, His 259, and His261, are shown as green rectangles.

a

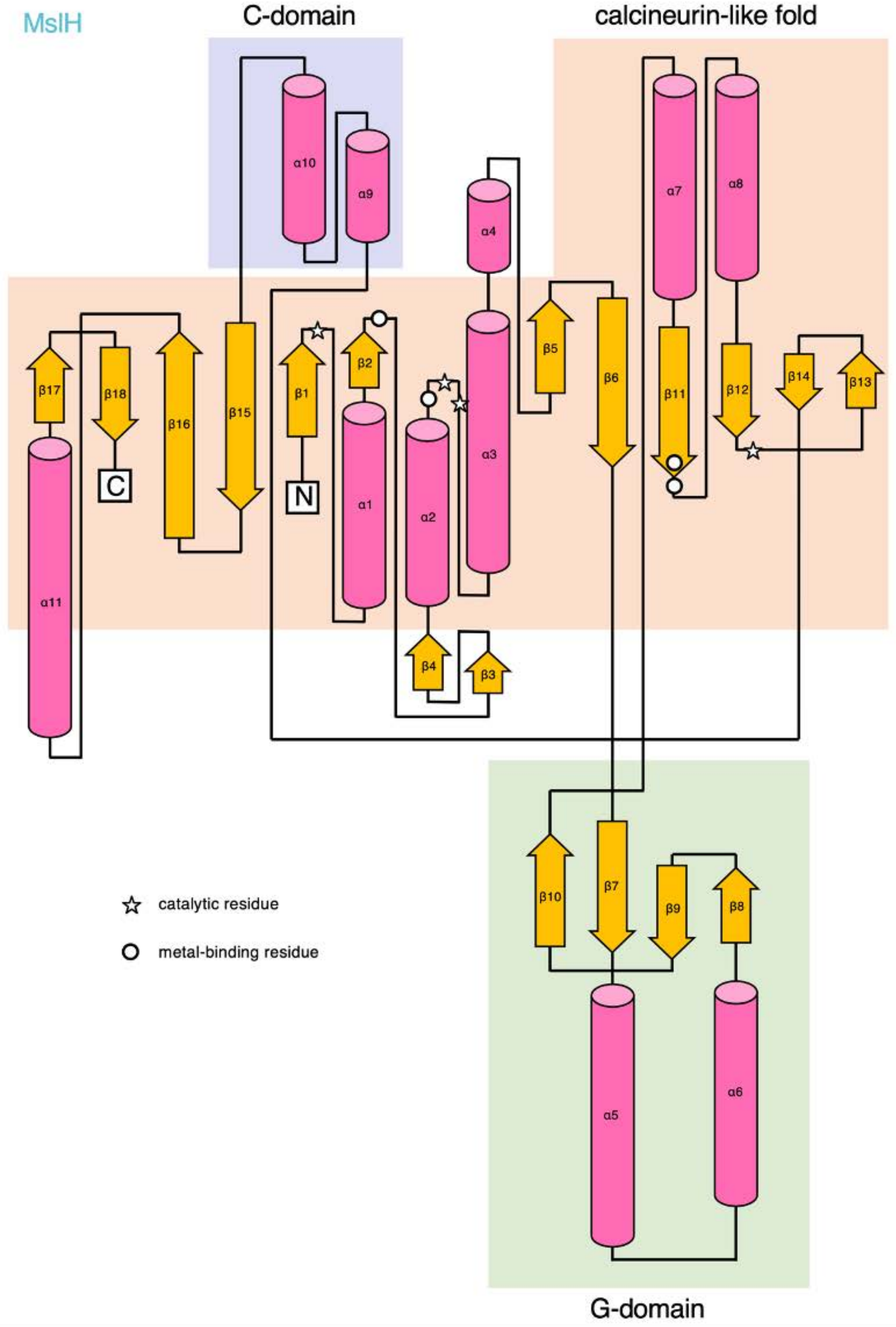
MslH
C-domain
calcineurin-like fold
G-domain
α10
α9
α4
α7
α8
α1
α2
α3
α11
α5
α6
β1
β2
β3
β4
β5
β6
β7
β8
β9
β10
β11
β12
β13
β14
β15
β16
β17
β18
N
C
catalytic residue
metal-binding residue

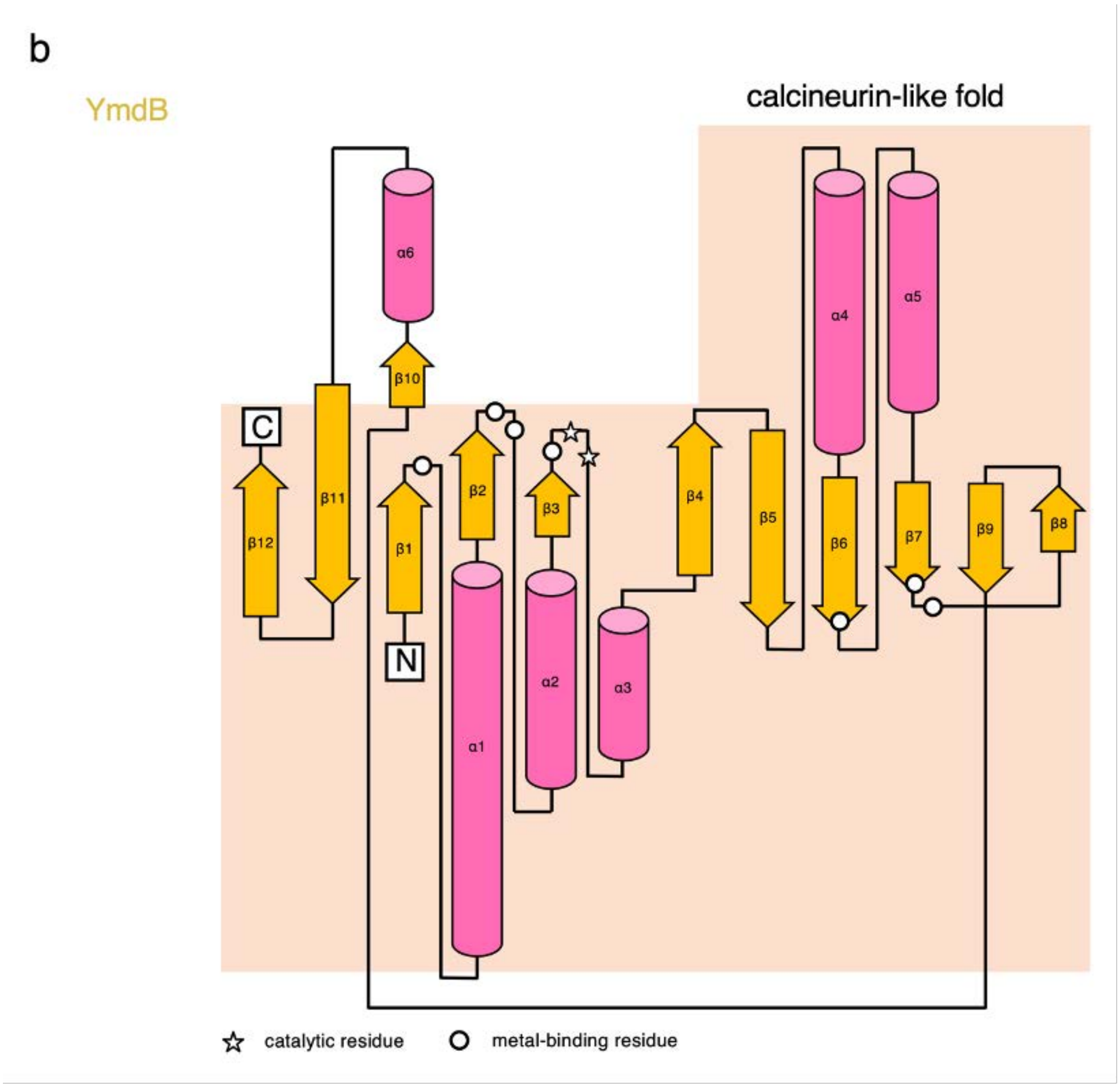

Supplementary Figure 6. Topology diagram of the MslH structure with the reported YmdB structure. Pink cylinders (α-helices) and yellow arrows (β-sheets) represent the secondary structural components of MslH **(a)** and YmdB **(b)**. Catalytic residues and metal-binding residues are symbolized as stars and circles, respectively. The C- and G-domains, the dimer forming motifs in MslH, are shown as purple and green boxes. The orange box shows the conserved calcineurin-like fold motif.

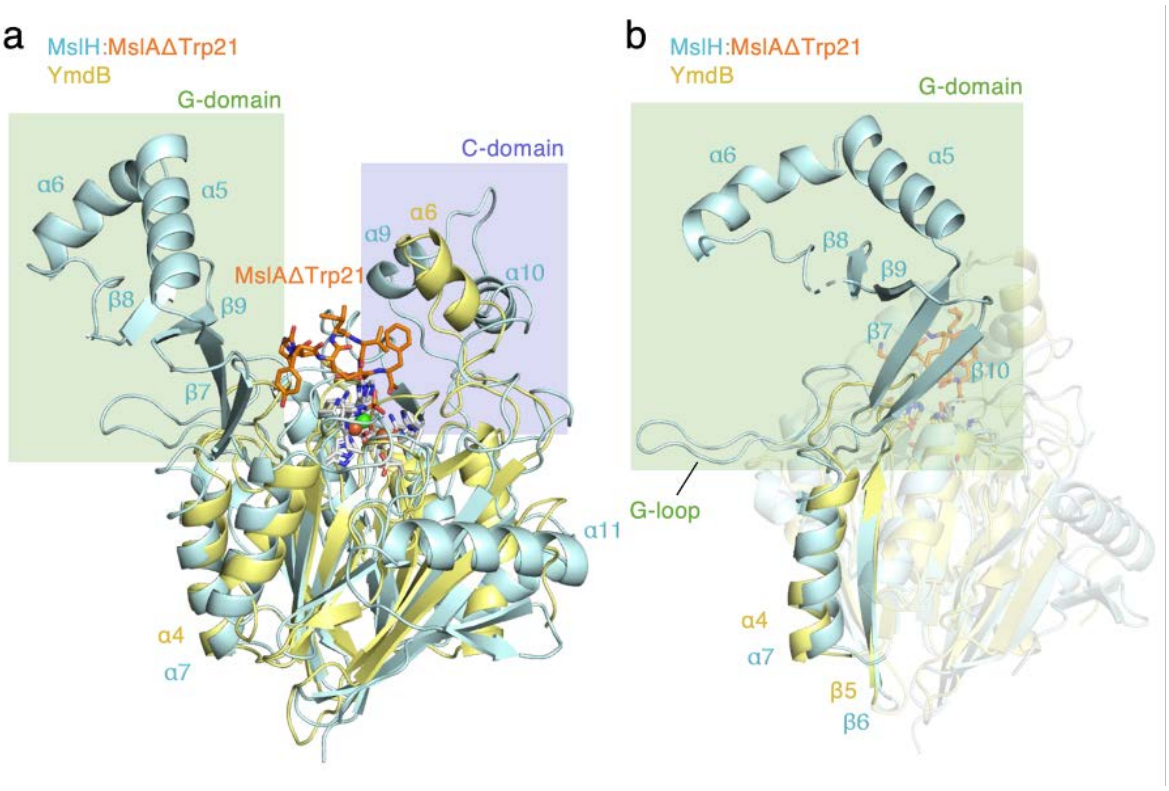

Supplementary Figure 7. Superimposition of the MslH:MslAΔTrp21 structure with the reported YmdB structure (PDB ID: 4B2O). (a) Superimposition of the MslH:MslAΔTrp21 structure (pale cyan-colored ribbon model) with the reported YmdB structure (yellow-colored ribbon model) reveals the formation of similar calcineurin-like folds. **(b)** Superimposition of views from different angles of panel **a**, focused on the characteristic architecture of MslH (from β6 to α7), including the G-domain.

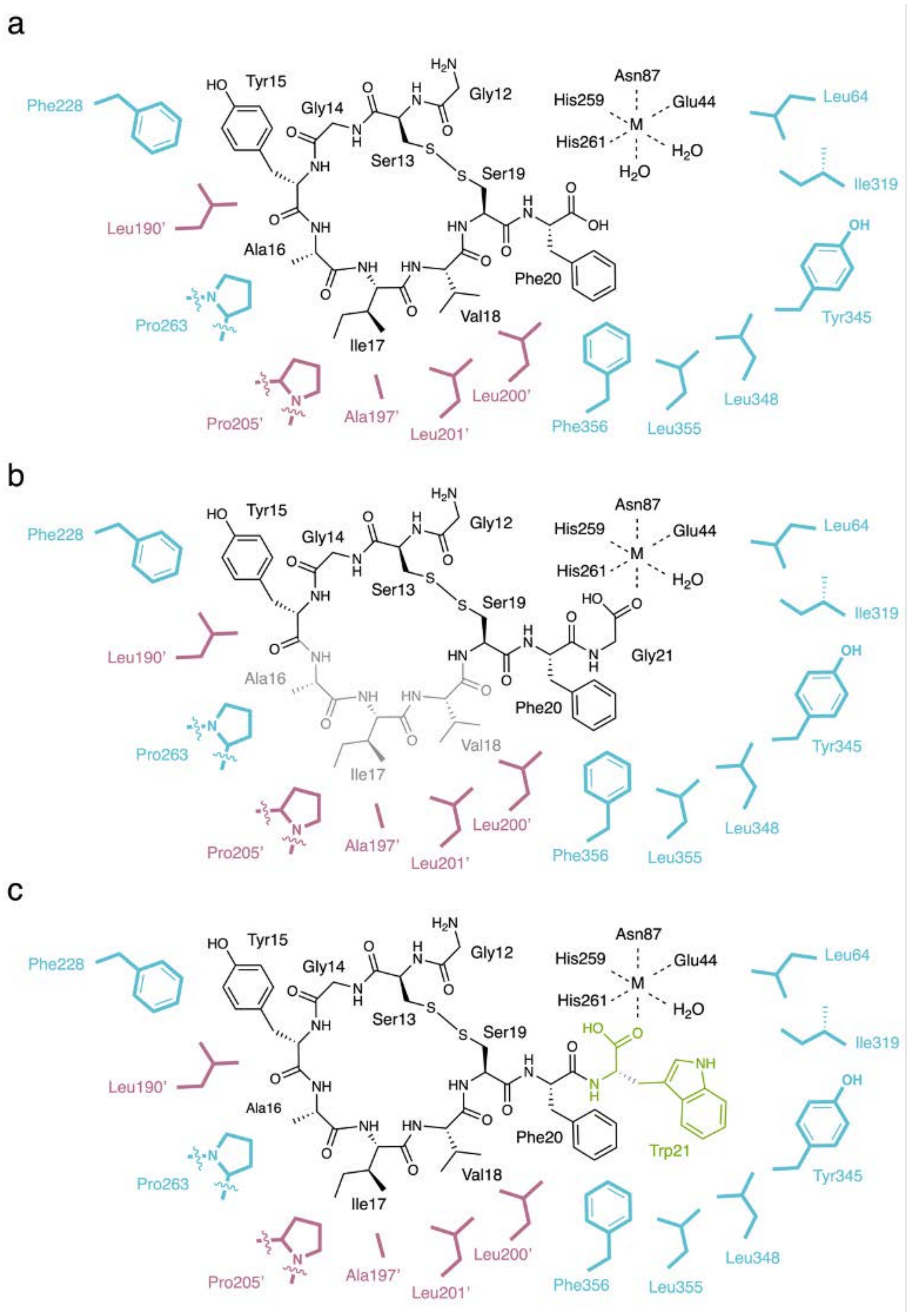

Supplementary Figure 8. Scheme of the binding mode of cMslA Gly12-Phe20 in the MslH:MslAΔTrp21 crystal structure (a), cMslA Gly12-Gly21 in the MslH:MslA Trp21G crystal structure (b), and modeled cMslA Gly12-Trp21 in the MslH:MslAΔTrp21 crystal structure (c). Color code: carbon-backbone of MslH in monomer A: cyan; carbon-backbone of MslH in monomer B: pink; carbon-backbone of the unclear residues of cMslA Gly12-Gly21 in the MslH:MslA Trp21G crystal structure: gray; carbon-backbone of modeled MslA Trp21: grass green.

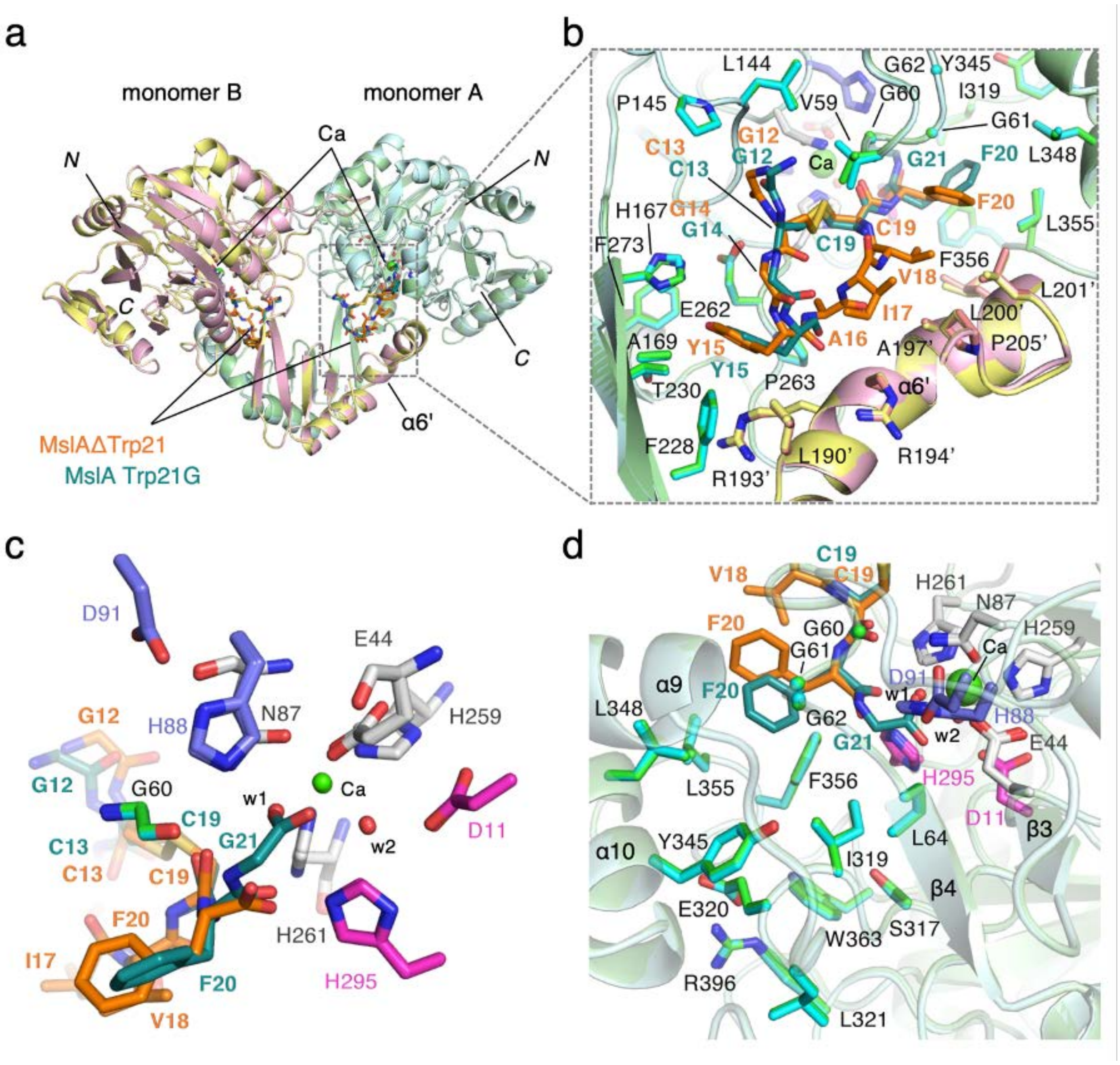

Supplementary Figure 9. Superimposition of the MslH:MslAΔTrp21 and MslH:MslA Trp21G structures. Color code: MslH:MslAΔTrp21 structure (MslH in monomer A: pale cyan; MslH in monomer B: light pink; carbon-backbone of MslH: cyan and pink; carbon-backbone of MslAΔTrp21: orange); MslH:MslA Trp21G structure (MslH in monomer A: pale green; MslH in monomer B: pale yellow; carbon-backbone of MslH: green and yellow; carbon-backbone of MslA Trp21G: blue green); Ca: light green; oxygen: red; nitrogen: blue; w: water molecule.

(a) Superimposition of the overall structure as the dimer (Cα RMSD = 0.11 Å). **(b)** Superimposition of close-up view of the MslH-MslA analogue binding cleft. **(c)** Superimposition of close-up views of the metal-coordination site. **(d)** Superimposed views of the hydrophobic pocket around the *C*-terminus of MslA analogues.

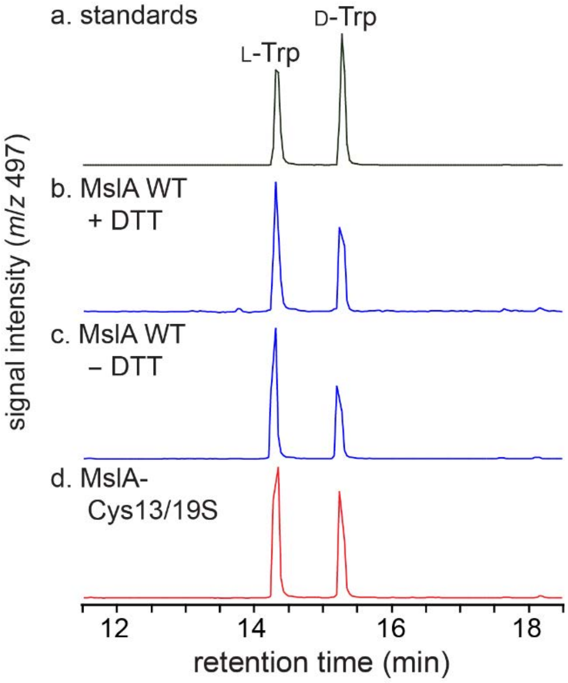

Supplementary Figure 10. LC-MS analysis of L-FDLA–Trp for the *in vitro* reaction of MslH. Data were acquired in the ESI negative ion mode. The *y*-axis represents signal strength measured at *m/z* 497, and the same scale applies to all chromatograms. **(a)** L-Trp and D-Trp standards. **(b)** Wild-type MslA (MslA WT) + MslH with DTT. **(c)** Wild-type MslA (MslA WT) + MslH without DTT. **(d)** MslA Cys13/19S variant + MslH with DTT.

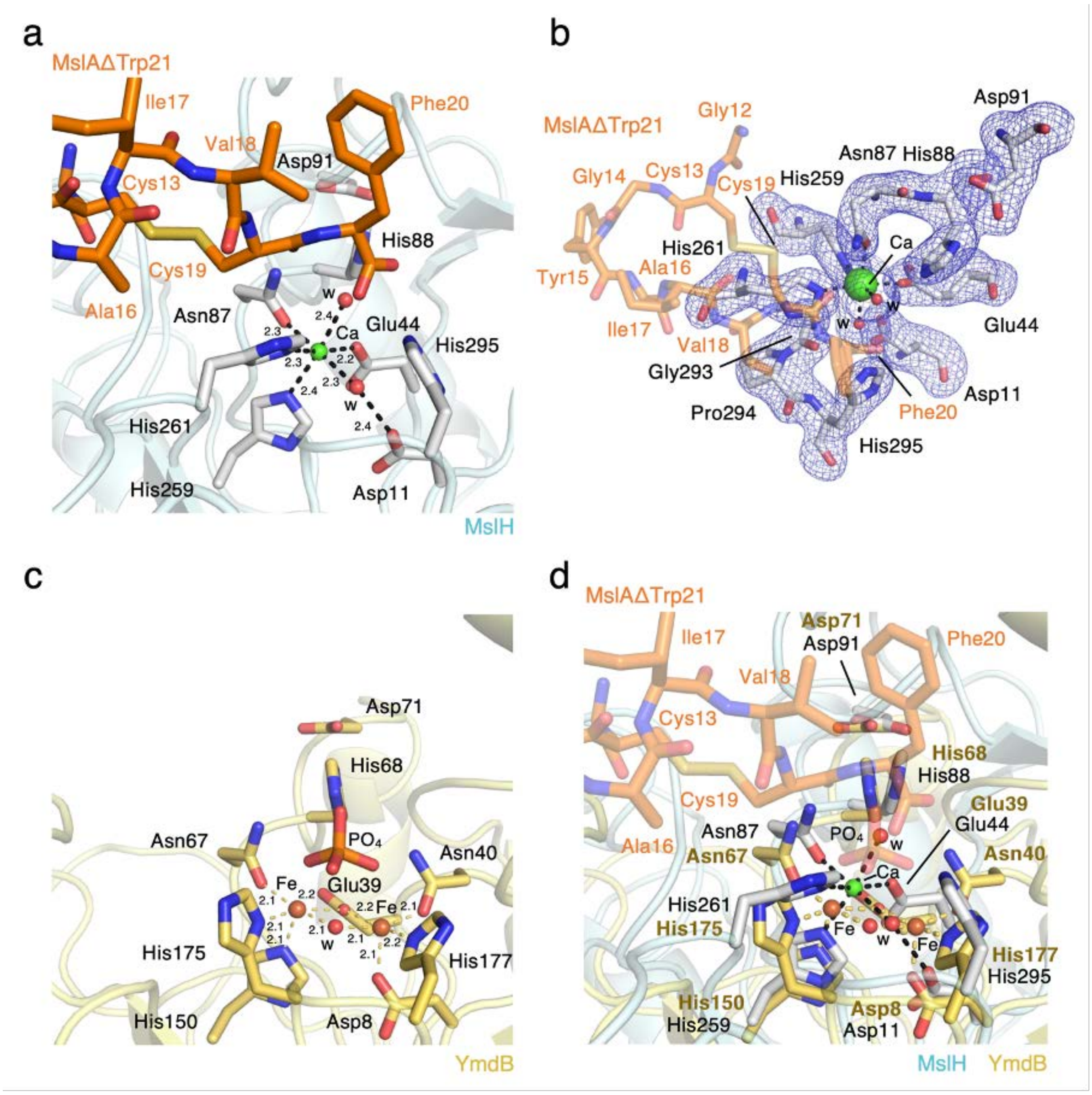

Supplementary Figure 11. Close-up views of the metal-binding sites in the MslH:MslAΔTrp21 structure (PDB ID: 8GQA) and the reported YmdB structure (PDB ID: 4B2O). (**a**) Close-up view of the MslH:MslAΔTrp21 crystal structure (MslH: pale cyan; carbon-backbone of MslH: white; carbon-backbone of MslAΔTrp21: orange; Ca: light green; w: water molecule). The dashed lines represent the distances in Å. (**b**) Representative OMIT electron density map (mF_o-DF_c) contoured to 3σ around Ca, with water molecules and MslH-active site residues shown in the blue-colored mesh. (**c**) Close-up view of the reported YmdB crystal structure (YmdB: pale yellow; carbon-backbone of YmdB: yellow; Fe: orange; w: water molecule; PDB ID: 4B2O). **(d)** Superimposition of the MslH:MslAΔTrp21 structure with the reported YmdB structure.

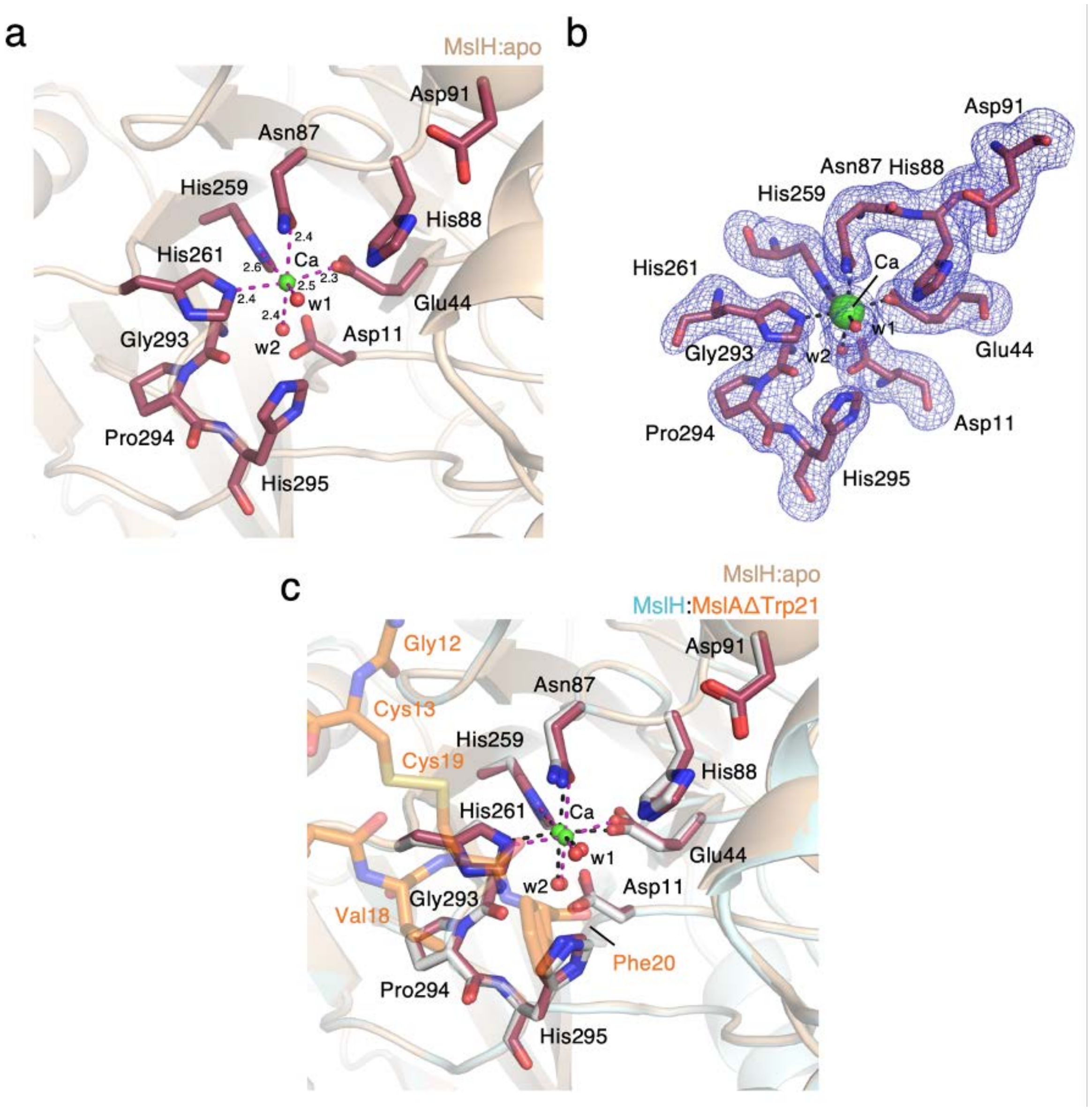

Supplementary Figure 12. Close-up views of the metal-binding sites in the MslH:apo structure (PDB ID: 8GQ9). (a) Close-up view of the MslH:apo crystal structure. The dashed lines represent the distances of metal-coordinating residues and water molecules (distances in Å). **(b)** Representative OMIT electron density map (mF_o-DF_c) contoured to 3σ around Ca. Water molecules and the MslH-active site residues are shown in the blue-colored mesh. **(c)** Superimposition of close-up views of the MslH:apo structure and the MslH:MslAΔTrp21 structure reveals nearly identical conformations of the side chains of MslH-active site residues.

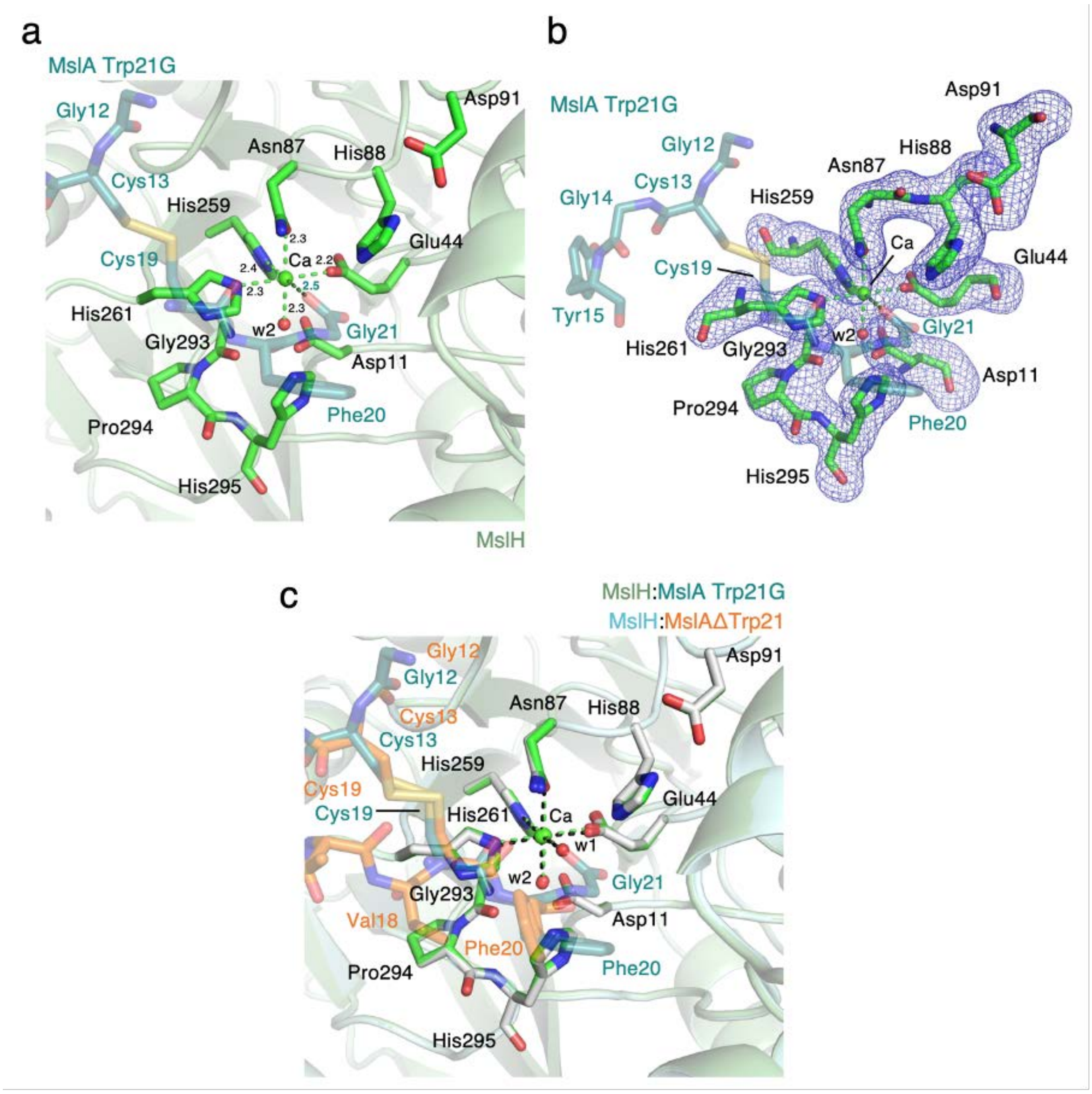

Supplementary Figure 13. Close-up views of the metal-binding sites in the MslH:MslA Trp21G structure (PDB ID: 8ITG). (a) Close-up view of the MslH:MslA Trp21G crystal structure. The dashed lines represent the distances of metal-coordinating residues and water molecules (distances in Å). **(b)** Representative OMIT electron density map (mF_o-DF_c) contoured to 3σ around Ca. Water molecules and the MslH-active site residues are shown in the blue-colored mesh. **(c)** Superimposition of close-up views of the MslH:MslA Trp21G structure and the MslH:MslAΔTrp21 structure, revealing the nearly identical conformations of the side chains of MslH-active site residues.

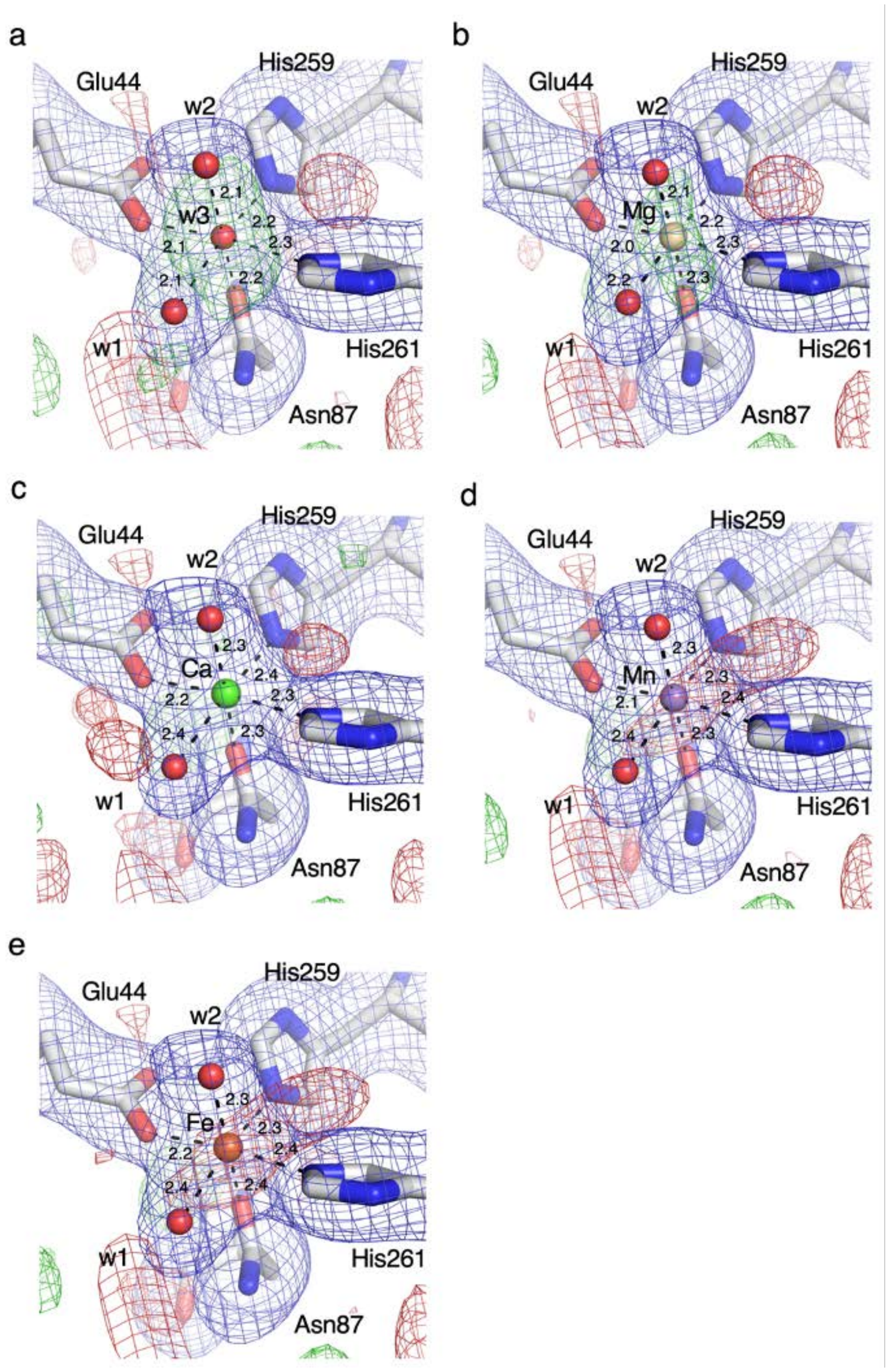

Supplementary Figure 14. Analysis of electron density maps for metals in the MslH:MslAΔTrp21 structure. Color code: carbon-backbone of MslH: white; Mg: wheat; Ca: light green; Mn: violet purple; Fe: orange; oxygen: red; nitrogen: blue; w: water molecule.

Representative OMIT electron density maps (mF_o-DF_c) contoured to 3σ around water molecules, metal-coordinated residues, and w3 **(a)**, Mg **(b)**, Ca **(c)**, Mn **(d)**, or Fe **(e)** are shown in the blue-colored mesh. Electron density maps (F_o-F_c) contoured to 2.2σ and -2.2σ around water molecules, metal-coordinated residues, and each metal ion are shown in green and red meshes, respectively. The dashed lines represent the distances of metal-coordinating residues and water molecules (distances in Å).

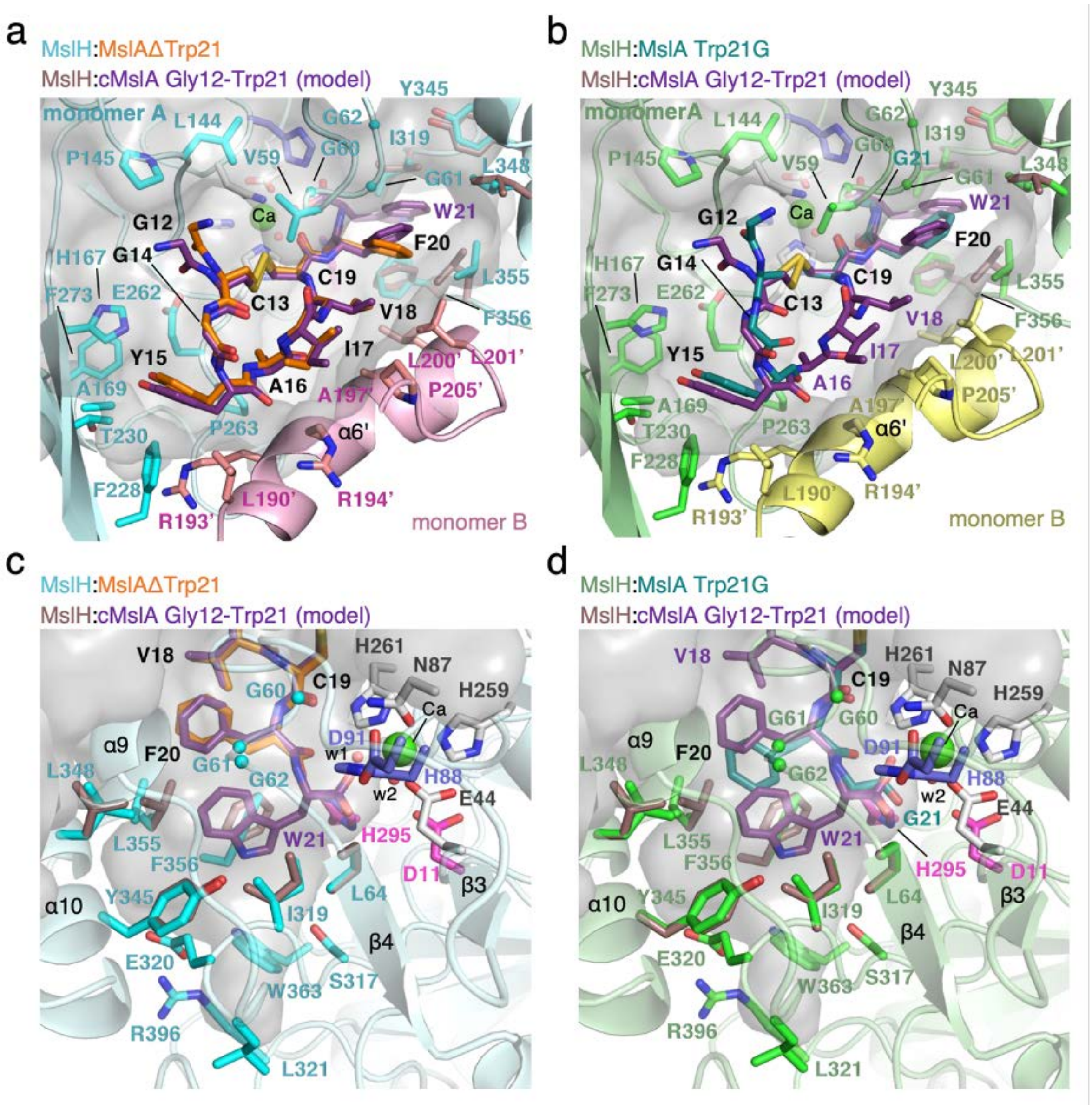

Supplementary Figure 15. Docking simulation of cMslA Gly12-Trp21 with surface image. Color code: MslH:MslAΔTrp21 structure (MslH in monomer A: pale cyan; MslH in monomer B: light pink; carbon-backbone of MslH: cyan and pink; carbon-backbone of MslAΔTrp21: orange); MslH:MslA Trp21G structure (MslH in monomer A: pale green; MslH in monomer B: pale yellow; carbon-backbone of MslH: green and yellow; carbon-backbone of MslA Trp21G: blue green); simulated cMslA Gly12-Trp21 in the MslH:MslAΔTrp21 structure (carbon-backbone of MslH: light purple; carbon-backbone of cMslA Gly12-Trp21: dark purple); Ca: light green; oxygen: red; nitrogen: blue; w: water molecule.

Superimposition of close-up views of the simulated cMslA Gly12-Trp21 with the MslH:MslAΔTrp21 structure **(a)** and the MslH:MslA Trp21G structure **(b)**. Superimpositions of the hydrophobic pocket around the *C*-terminus of cMslA Gly12-Trp21 with the MslH:MslAΔTrp21 structure **(c)** and the MslH:MslA Trp21G structure **(d)**. The surface models are from the docking-simulated MslH structure.

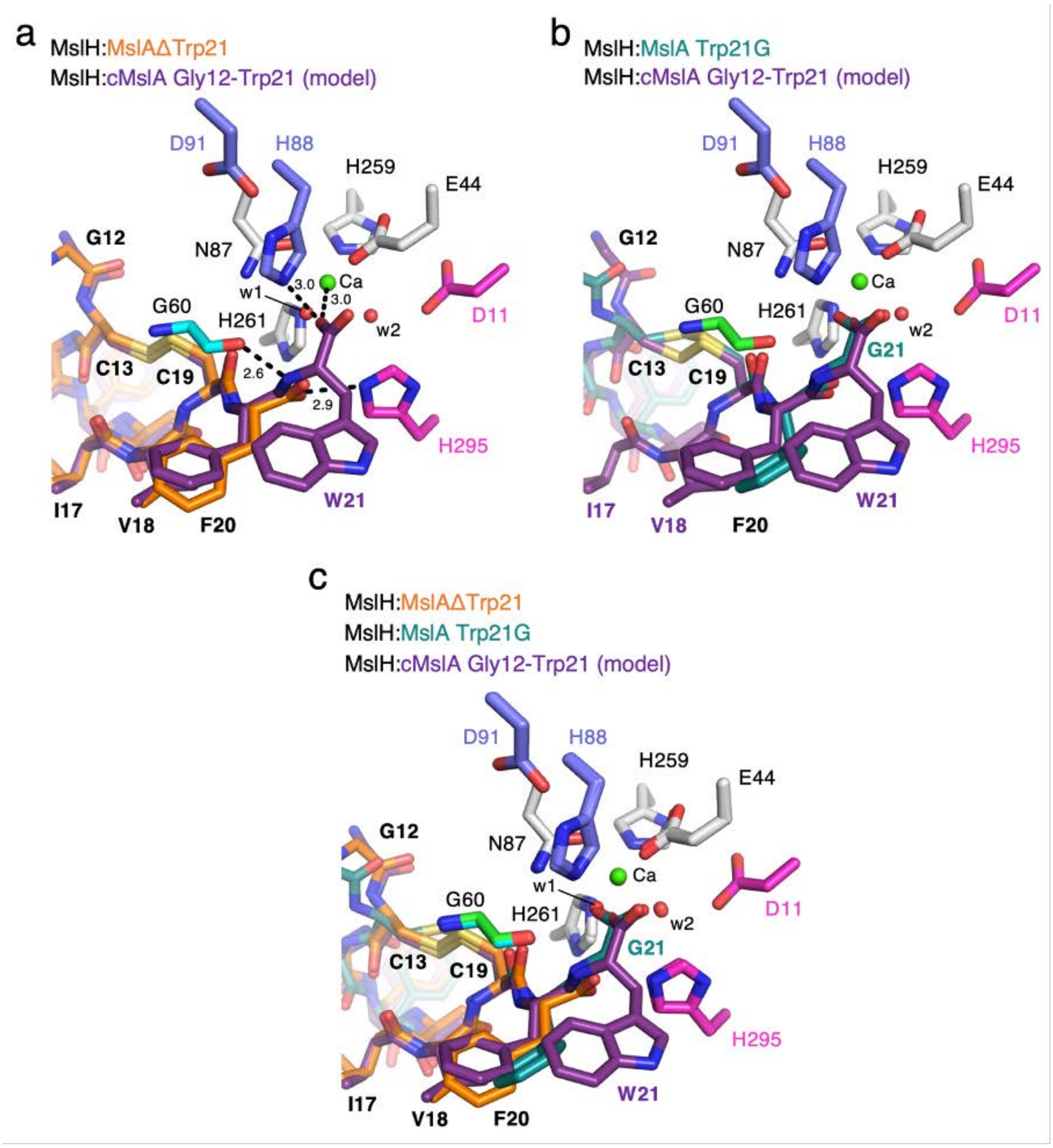

Supplementary Figure 16. Close-up view from the docking-simulated cMslA Gly12-Trp21 around the *C*-terminus of MslA. Color code: carbon-backbone of MslAΔTrp21: orange; carbon-backbone of MslA Trp21G: blue green; carbon-backbone of simulated cMslA Gly12-Trp21: dark purple; Ca: light green; oxygen: red; nitrogen: blue; w: water molecule.

Superimposition of close-up views of the simulated cMslA Gly12-Trp21 with the MslH:MslAΔTrp21 structure **(a)**, the MslH:MslA Trp21G structure **(b)**, and the MslH:MslAΔTrp21/MslH:MslA Trp21G structures **(c)**. The dashed lines represent the distances in Å. The nitrogen and oxygen atoms forming the peptide bond between MslA Phe20 and Trp21 interacted with MslH via polar interactions with the oxygen atom of the backbone of MslH Gly60 and the nitrogen atom of the His295 side chain, respectively (2.6 Å and 2.9 Å). The oxygen atom of the carboxyl group of Trp21 interacted with MslH His88 and Ca(II) ion at distances of 3.0 Å and 3.0 Å, respectively.

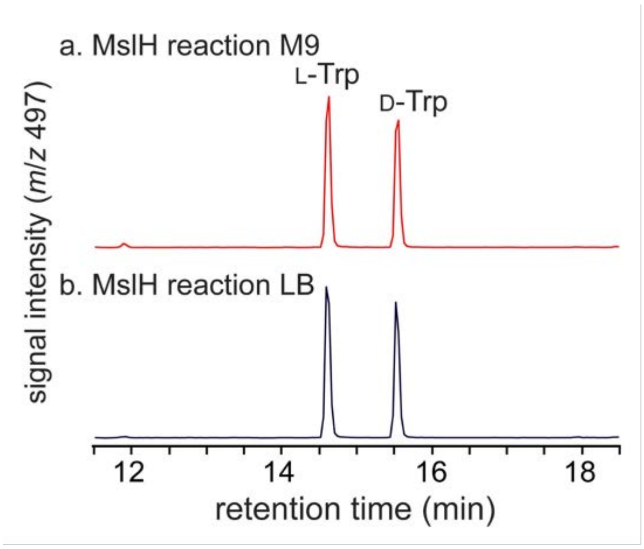

Supplementary Figure 17. LC-MS analysis of L-FDLA-Trp for the *in vitro* reactions of MslH expressed in cells grown in M9 and LB media. Data were acquired in the ESI negative ion mode. The *y*-axis represents signal strength measured at *m/z* 497, and the same scale applies to all chromatograms. **(a)** Reaction with MslH expressed from M9 medium and **(b)** reaction with MslH expressed from LB medium.

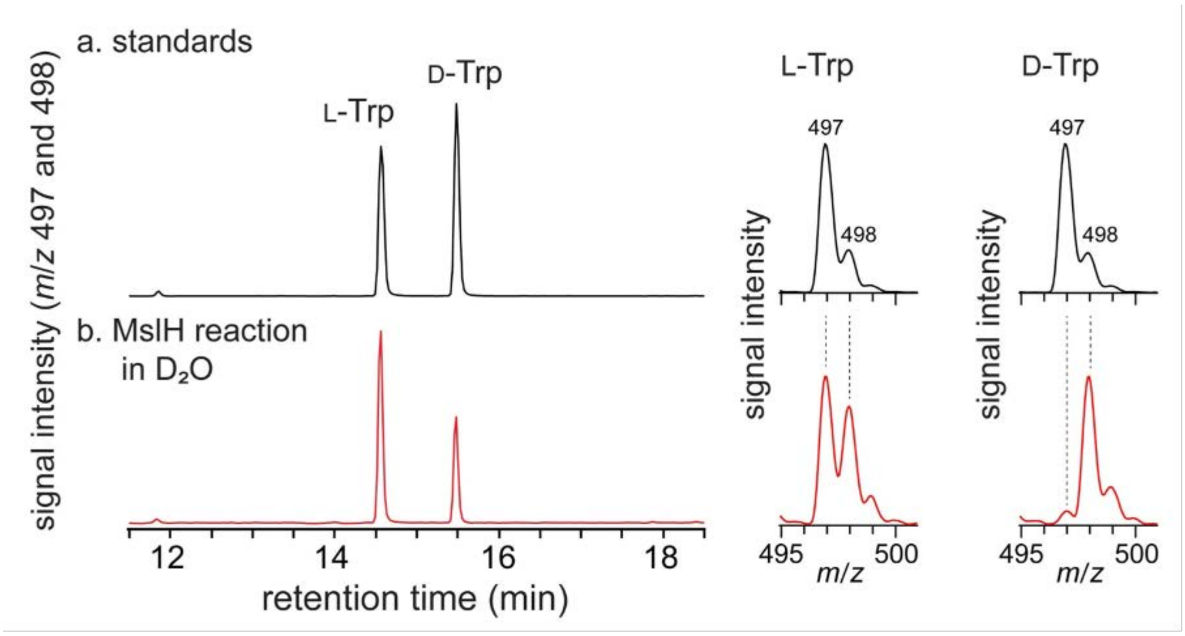

Supplementary Figure 18. LC-MS analysis of L-FDLA-Trp for the *in vitro* reaction of MslH in heavy water. Data were acquired in the ESI negative ion mode. Chromatogram monitored with *m*/*z* 496.5–498.5 (left) and mass spectra (right) at retention time of 14.5 min (for L-Trp) and 15.5 min (for D-Trp) of **(a)** authentic standards of a mixture of L,D-Trp and **(b)** Trp from the MslH reaction in D_2O.

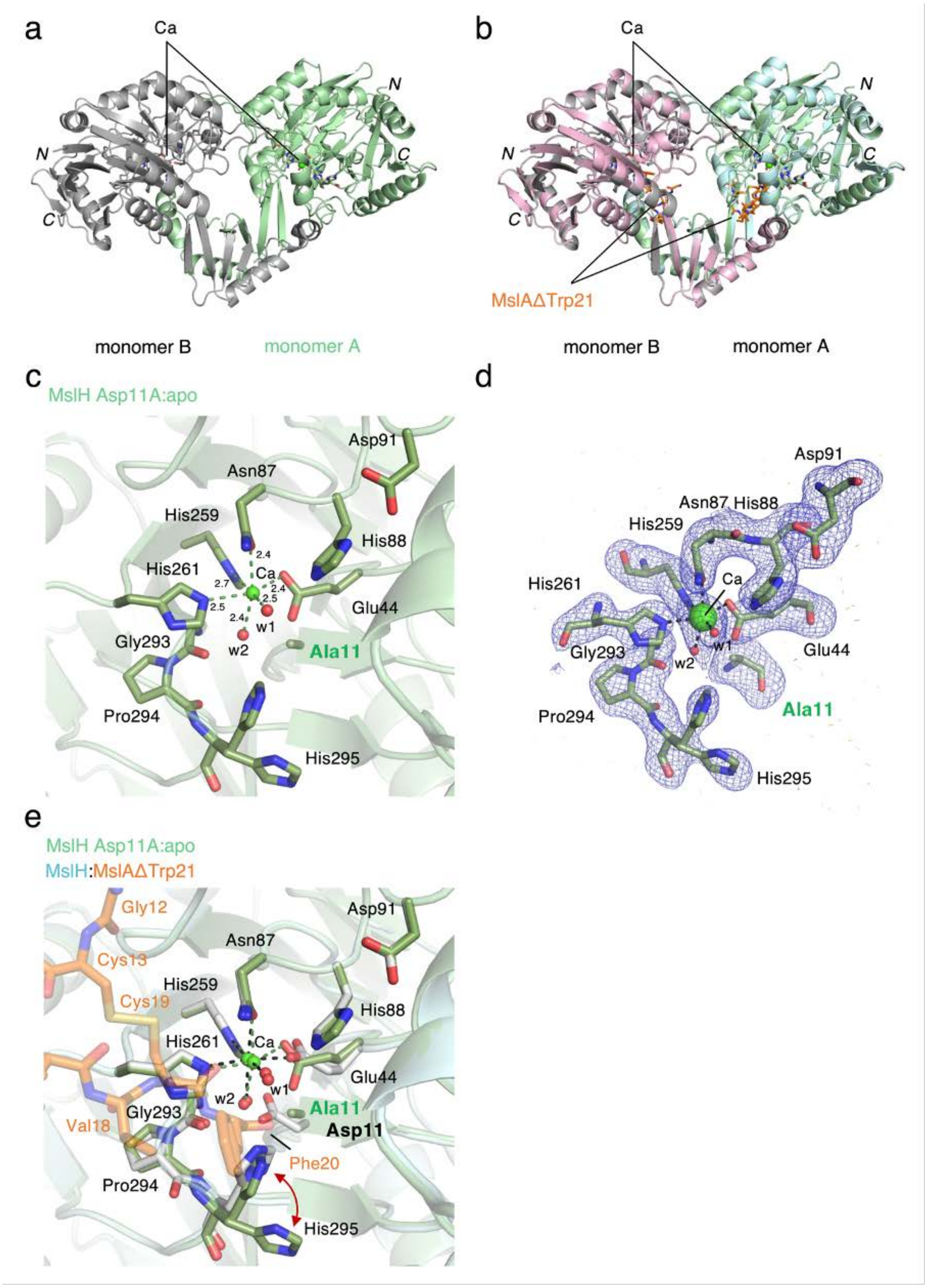
a
Ca
N
C
N
C
monomer B
monomer A
b
Ca
N
N
C
C
MslAΔTrp21
monomer B
monomer A
c
MslH Asp11A:apo
Asp91
Asn87
His259
His88
Ca
His261
Glu44
w1
w2
Gly293
Ala11
Pro294
His295
d
Asp91
Asn87 His88
His259
Ca
His261
Gly293
w1
w2
Glu44
Ala11
Pro294
His295
e
MslH Asp11A:apo
MslH:MslAΔTrp21
Gly12
Asp91
Cys13
Asn87
His259
Cys19
His88
His261
Ca
Glu44
w1
w2
Ala11
Asp11
Val18
Gly293
Phe20
Pro294
His295

Supplementary Figure 19. Views of the structure of MslH Asp11A:apo (PDB ID: 8GQB). Color code: MslH Asp11A in monomer A: light green; MslH Asp11A in monomer B: grey; carbon-backbone of MslH Asp11A: dark green; Ca: light green; oxygen: red; nitrogen: blue; w: water molecule.

(**a**) Overview of the dimer formation in the MslH Asp11A:apo crystal structure. (**b**) Superimposition of the MslH Asp11A:apo structure with the MslH:MslAΔTrp21 structure (MslH in monomer A: pale cyan; MslH in monomer B: light pink; carbon-backbone of MslH: white; carbon-backbone of MslAΔTrp21: orange) reveals similar MslH conformations (Cα RMSD = 0.16 Å). **(c)** Close-up view of the MslH Asp11A:apo crystal structure. The dashed lines represent the distances of metal-coordinating residues and water molecules (distances in Å). **(d)** Representative OMIT electron density map (mF_o-DF_c) contoured to 3σ around Ca; water molecules and MslH Asp11A-active site residues are shown in the blue-colored mesh. **(e)** Superimposed close-up views of the MslH Asp11A:apo structure with the MslH:MslAΔTrp21 structure reveal the nearly identical conformations of the side chains of MslH-active site residues, including the metal coordinating residues, as well as the alternative structures of His295 residues, the 180 degree flip of the Pro294 residue main chain, and the 96 degree flip of the Glu44 side chain.

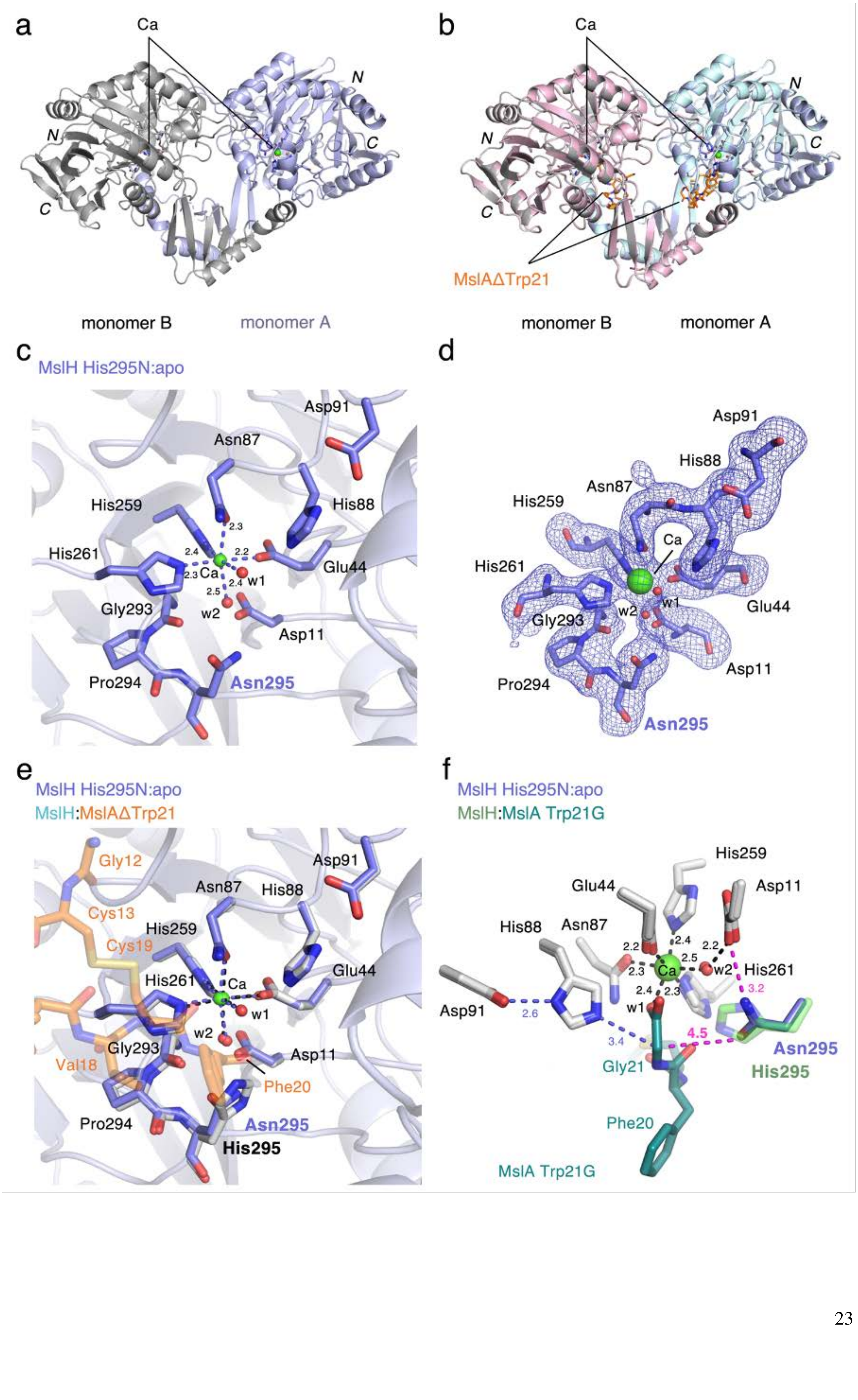
a
Ca
N
N
C
C
monomer B
monomer A
b
Ca
N
N
C
C
MslAΔTrp21
monomer B
monomer A
c
MslH His295N:apo
Asp91
Asn87
His259
His88
His261
Ca
w1
Glu44
Gly293
w2
Asp11
Pro294
Asn295
d
Asp91
His88
Asn87
His259
Ca
His261
w1
Glu44
w2
Gly293
Asp11
Pro294
Asn295
e
MslH His295N:apo
MslH:MslAΔTrp21
Gly12
Asp91
Asn87
His88
Cys13
His259
Cys19
Glu44
His261
Ca
w1
w2
Gly293
Asp11
Val18
Phe20
Pro294
Asn295
His295
f
MslH His295N:apo
MslH:MslA Trp21G
His259
Glu44
Asp11
His88
Asn87
Ca
w2
His261
Asp91
w1
Asn295
His295
Gly21
Phe20
MslA Trp21G

Supplementary Figure 20. Views of the structure of MslH His295N:apo (PDB ID: 8ITH). Color code: MslH His295N in monomer A: light blue; MslH His295N in monomer B: grey; carbon-backbone of MslH His295N: blue; Ca: light green; oxygen: red; nitrogen: blue; w: water molecule.

(**a**) Overview of the dimer formation in the MslH His295N:apo crystal structure. (**b**) Superimposition of the MslH His295N:apo structure with the MslH:MslAΔTrp21 structure (MslH in monomer A: pale cyan; MslH in monomer B: light pink; carbon-backbone of MslH: white; carbon-backbone of MslAΔTrp21: orange) reveals similar MslH conformations (Cα RMSD = 0.07 Å). **(c)** Close-up view of the MslH His295N:apo crystal structure. The dashed lines represent the distances of metal-coordinating residues and water molecules (distances in Å). **(d)** Representative OMIT electron density map (mF_o-DF_c) contoured to 2.5σ around Ca; water molecules and MslH His295N-active site residues are shown in the blue-colored mesh. **(e)** Superimposed close-up views of the MslH His295N:apo structure with the MslH:MslAΔTrp21 structure reveal the nearly identical conformations of the side chains of MslH-active site residues, including the metal coordinating residues. **(f)** Superimposed close-up views of the MslH His295N:apo structure with the MslH:MslA Trp21G structure reveal the nearly identical conformations around the *C*-terminal MslA Gly21 binding site.

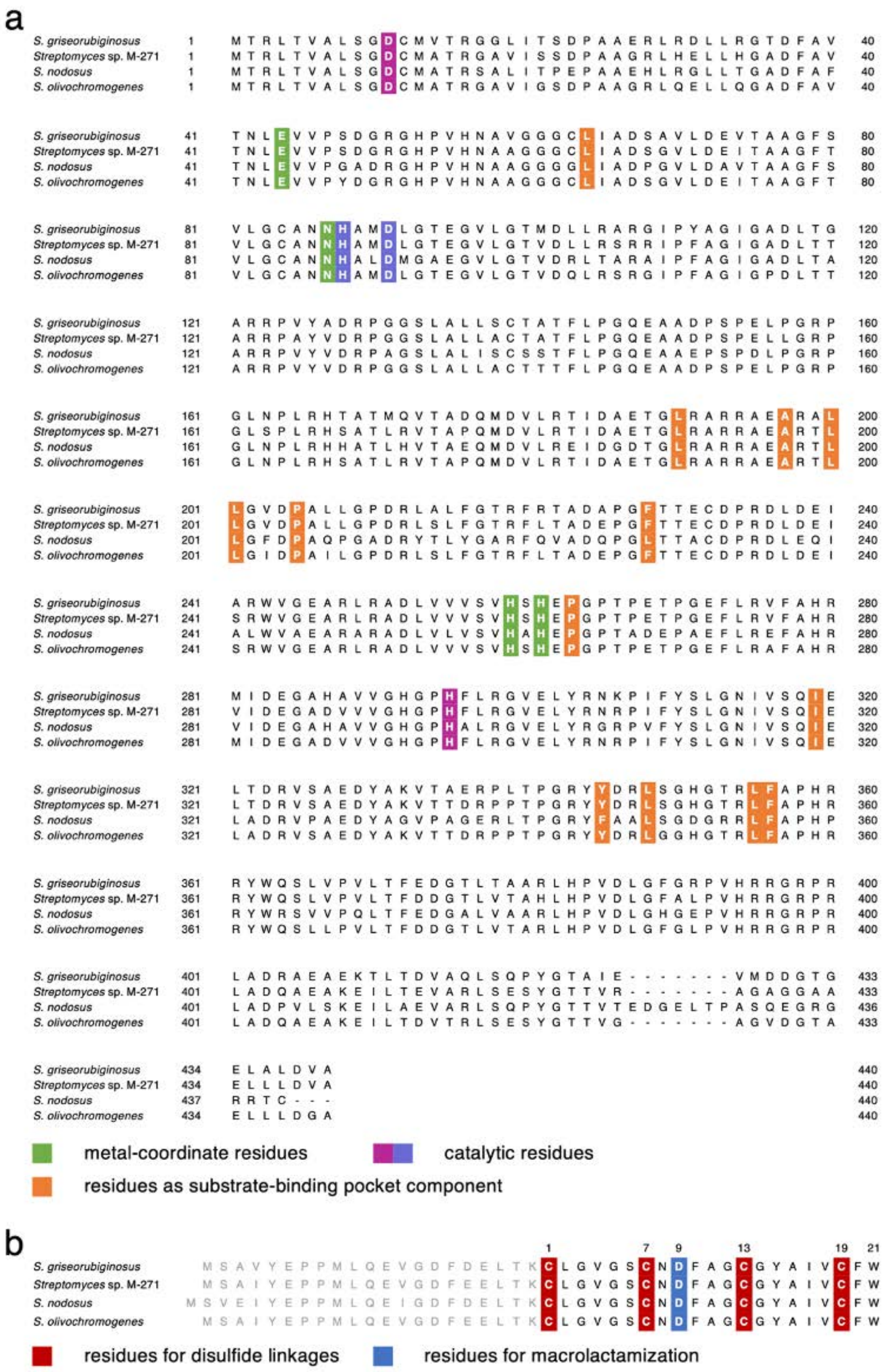

Supplementary Figure 21. Sequence alignment of MslH and MslA. (a) The MslH sequence derived from *Streptomyces griseorubiginosus* NBRC12899 in this study was aligned with homologous MslH-like enzymes derived from *Streptomyces* sp. M-271 (86% amino acid identity), *Streptomyces nodosus strain* ATCC 14899 (72% amino acid identity), and *Streptomyces olivochromogenes* NBRC 3561 (85% amino acid identity). **(b)** Sequence alignment of MslA. The leader peptide is colored gray.

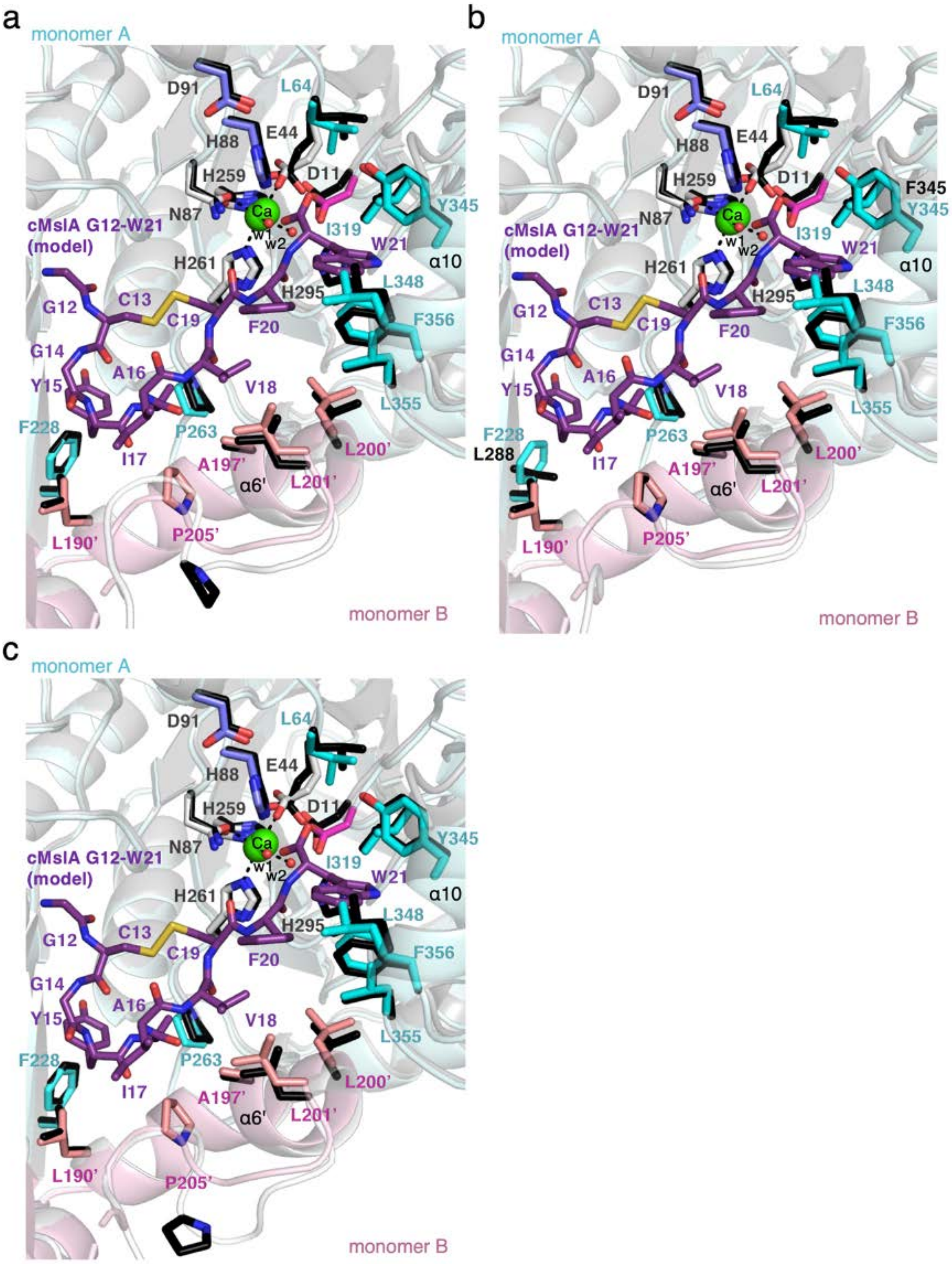

Supplementary Figure 22. Model analysis of MslH-like enzymes. Superimposed views of the MslH crystal structure (MslH in monomer A: pale cyan; MslH in monomer B: light pink; carbon-backbone of MslH: cyan and pink) with modeled MslH-like enzyme structures (MslH: gray, carbon-backbone of stick models: black) derived from **(a)** *Streptomyces* sp. M-271 (Cα RMSD = 0.17 Å), **(b)** *S. nodosus* ATCC 14899 (Cα RMSD = 0.18 Å), and **(c)** *S. olivochromogenes* NBRC 3561 (Cα RMSD = 0.17 Å). The docking-simulated cMslA Gly12-Trp21 structure (stick model of carbon-backbone: dark purple) was superimposed.

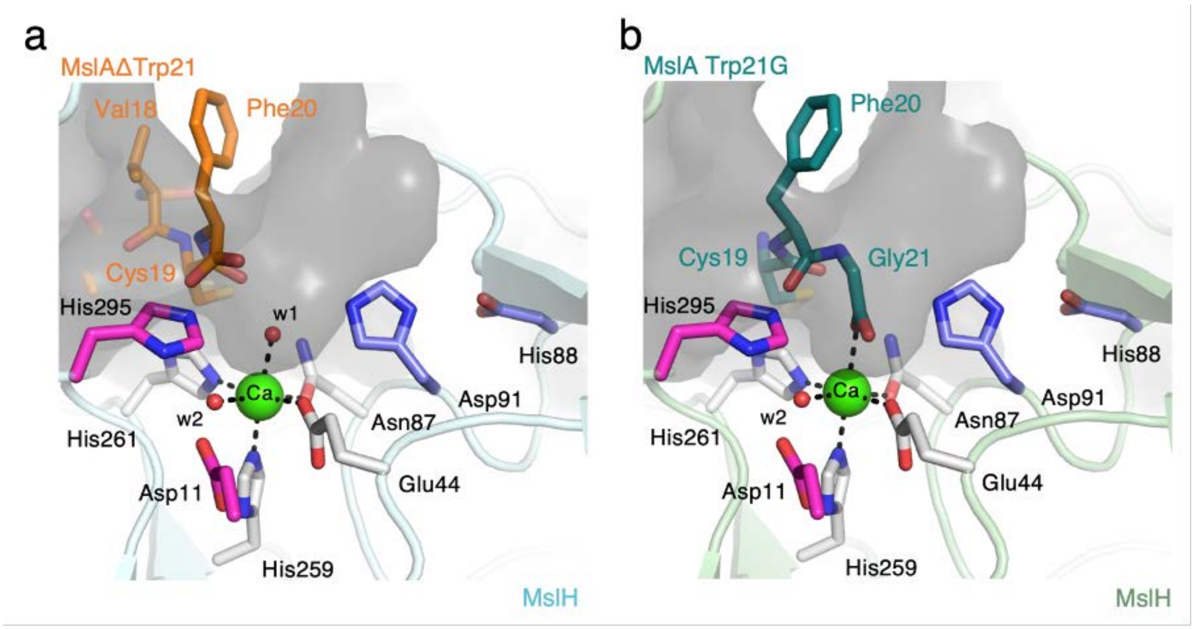

Supplementary Figure 23. Surface views of the metal-binding site. Surface models of the metal-binding site in the MslH:MslAΔTrp21 structure **(a)** and MslH:MslA Trp21G structure **(b)**. The sphere radius for Ca was set as 1.0 Å, which is the same as the ionic radius of the Ca(II) ion. The dashed lines represent the distances in Å.

2. Supplementary tables

Supplementary Table 1. Crystallization conditions, data collection, and refinement statistics.[a]

	MslH:apo	MslH:MslAΔTrp21	MslH:MslA Trp21G
**PDB ID**	8GQ9	8GQA	8ITG
**Crystallization**			
Precipitation conditions	5.00 mg/mL MslH, 200 mM magnesium chloride, 100 mM Tris-HCl (pH 8.0), 28% (w/v) PEG400	5.00 mg/mL MslH, 1.89 mg/mL MslAΔTrp21-MslB1, 200 mM magnesium chloride, 100 mM Tris-HCl (pH 9.0), 28% (w/v) PEG400	5.00 mg/mL MslH, 1.89 mg/mL MslA Trp21G-MslB1, 200 mM magnesium chloride, 100 mM Tris-HCl (pH 9.0), 34% (w/v) PEG400
**Data collection**			
Space group	*I*422	*I*422	*I*422
Cell dimensions:			
a, *b*, *c* (Å)	128.02, 128.02, 170.59	128.03, 128.03, 171.03	127.79, 127.79, 170.67
α, β, γ (°)	90.00, 90.00, 90.00	90.00, 90.00, 90.00	90.00, 90.00, 90.00
X-Ray source[b]	Synchrotron (PF BL-1A)	Synchrotron (PF BL-1A)	Synchrotron (PF BL-1A)
Resolution (Å)[c]	45.26-2.30 (2.38-2.30)	45.27-2.29 (2.38-2.29)	45.18-2.12 (2.18-2.12)
R_{merge}	0.259 (2.225)	0.155 (1.760)	0.080 (0.821)
I / σI	20.3 (2.7)	19.0 (2.3)	18.7 (2.3)
CC (1/2)	0.999 (0.822)	0.999 (0.867)	0.999 (0.810)
Total number of reflections	1610820 (132719)	895893 (96436)	304810 (25701)
Total number unique reflections	31782 (3075)	32290 (3336)	40284 (3264)
Completeness (%)	100.0 (98.9)	100.0 (100.0)	100.0 (100.0)
Multiplicity	50.7 (43.2)	27.7 (28.9)	7.6 (7.9)
**Refinement**			
R_{work} / R_{free}	0.177 / 0.205	0.184 / 0.208	0.182 / 0.207
No. atoms:	3582	3629	3627
B-factors:	49.9	47.6	52.8
R.m.s. deviations:			
Bond lengths (Å)	0.003	0.002	0.003
Bond angles (°)	0.539	0.555	0.542

[a)]Experimental details are described in the Methods section (Crystallography). [b)]PF: Photon Factory. [c)]Values in parentheses are for highest-resolution shell.

Supplementary Table 2. Crystallization conditions, data collection, and refinement statistics.[a]

	MslH Asp11A:apo	MslH His295N:apo
**PDB ID**	8GQB	8ITH
**Crystallization**		
Precipitation conditions	5.00 mg/mL MslH Asp11A, 200 mM magnesium chloride, 100 mM Tris-HCl (pH 8.5), 20% (w/v) PEG400	5.00 mg/mL MslH His295N, 200 mM magnesium chloride, 100 mM Tris-HCl (pH 9.0), 28% (w/v) PEG400
**Data collection**		
Space group	*I*422	*I*422
Cell dimensions:		
a, *b*, *c* (Å)	127.11, 127.11, 170.65	127.89, 127.89, 171.29
α, β, γ (°)	90.00, 90.00, 90.00	90.00, 90.00, 90.00
X-Ray source[b]	Synchrotron (PF BL-1A)	Synchrotron (PF BL-1A)
Resolution (Å)[c]	44.94-2.41 (2.50-2.41)	45.22-2.55 (2.66-2.55)
R_{merge}	0.142 (1.425)	0.112 (0.934)
$I / \sigma I$	16.3 (2.2)	15.1 (2.3)
CC (1/2)	0.999 (0.860)	0.997 (0.787)
Total number of reflections	444381 (43222)	175908 (21970)
Total number unique reflections	27340 (2833)	23055 (2797)
Completeness (%)	100.0 (100.0)	98.7 (99.6)
Multiplicity	16.3 (15.3)	7.6 (7.9)
**Refinement**		
R_{work} / R_{free}	0.195 / 0.223	0.183 / 0.216
No. atoms:	3537	3480
B-factors:	56.7	50.7
R.m.s. deviations:		
Bond lengths (Å)	0.002	0.002
Bond angles (°)	0.487	0.507

[a)]Experimental details are described in the Methods section (Crystallography). [b)]PF: Photon Factory. [c)]Values in parentheses are for highest-resolution shell.

Supplementary Table 3. ICP-MS measurements (continues on the following page).

	24 Mg [He]		24 -> 24 Mg [H2]		27 Al [He]	
Sample Name	CPS	CPS RSD	CPS	CPS RSD	CPS	CPS RSD
standard 0ppb	13.33666667	43.29044758	34.44333333	5.598615791	3.333333333	173.2050808
standard 0.5ppb	131.1166667	21.17184458	946.7233333	1.863426324	41.11333333	32.76910036
standard 1ppb	184.4533333	8.148920686	1839.036667	4.174549656	54.44666667	88.36279772
standard 2ppb	416.69	9.08711798	3764.95	2.800991454	131.12	14.00257967
standard 5ppb	1047.843333	6.615026624	9360.376667	1.797868153	237.79	14.38770205
standard 10ppb	1881.27	3.464566396	19147.91333	2.883466961	556.6933333	5.712533463
standard curve	y = 188.57 * x + 31.02		y = 1911.24 * x - 44.08		y = 53.74 * x + 5.05	
HNO3 blank	46.67	21.42704093	664.48	2.474676639	6.666666667	132.325366
H2O blank	5.553333333	69.34441108	23.33333333	65.46536707	4.443333333	114.6012337
MsIH	135.5633333	5.117572895	1519.003333	12.52044736	94.44666667	10.18421912
	Conc. (μg/mL)	Conc. RSD	Conc. (μg/mL)	Conc. RSD	Conc. (μg/mL)	Conc. RSD
Conc. in treated MsIH (ng/mL)	0.554371349	6.636377814	0.81783609	12.16739435	1.663499253	10.75942363
Conc. in original MsIH (μM)	0.006		0.008		0.015	

	27 -> 27 Al [H2]		40 -> 40 Ca [H2]		43 -> 43 Ca [H2]	
Sample Name	CPS	CPS RSD	CPS	CPS RSD	CPS	CPS RSD
standard 0ppb	37.77666667	10.19392825	462.2433333	11.74654096	3.333333333	100.0500375
standard 0.5ppb	1103.406667	1.6822637	4045.036667*	8.923349434*	15.55333333	68.89750727
standard 1ppb	1856.816667	6.672133595	5943.353333	3.817376553	6.666666667	132.325366
standard 2ppb	4122.803333	4.027481311	11660.61333	1.456253669	33.33666667	36.06161393
standard 5ppb	9858.446667	4.620632516	28827.11	2.721108944	88.89	11.45588708
standard 10ppb	20147.94333	2.358642289	55791.78667	0.246279551	170.0033333	17.97053271
standard curve	y = 2007.29 * x - 1.28		y = 5546.80 * x + 568.53		y = 17.02 * x + 0.50	
HNO3 blank	128.8933333	21.5370463	6102.316667	2.448813839	15.55666667	32.73268579
H2O blank	16.66666667	39.99000375	332.24	6.451765885	3.333333333	100.0500375
MsIH	3113.7	3.345296052	24709.06667	1.138729541	62.22666667	29.50630158
	Conc. (μg/mL)	Conc. RSD	Conc. (μg/mL)	Conc. RSD	Conc. (μg/mL)	Conc. RSD
Conc. in treated MsIH (ng/mL)	1.551832095	3.343926287	4.352153029	1.165547455	3.627666221	29.74317475
Conc. in original MsIH (mM)	0.014		0.027		0.023	

	44 -> 44 Ca [H2]		54 -> 54 Fe [H2]		56 Fe [He]	
Sample Name	CPS	CPS RSD	CPS	CPS RSD	CPS	CPS RSD
standard 0ppb	17.78	57.27299226	530.0266667	4.357141295	1243.426667	5.630063772
standard 0.5ppb	201.12	2.52916618	904.5066667	4.549122573	3098.15	2.060083435
standard 1ppb	304.46	6.320398237	1378.99	3.35278049	5093.093333	2.753457861
standard 2ppb	562.2533333	2.24492125	2143.54	2.32226753	9046.953333	0.788259326
standard 5ppb	1336.77	6.469786052	4837.473333	1.495855171	20078.01	2.823194335
standard 10ppb	2521.37	4.398139806	8951.356667	1.736347871	38919.03667	1.674471486
standard curve	y = 248.38 * x + 58.11		y = 847.16 * x + 512.23		y = 3762.19 * x + 1313.01	
HNO3 blank	216.6766667	9.358928898	307.79	12.45883581	1031.18	6.32169712
H2O blank	12.22	95.78184823	130.0033333	4.441042044	741.16	4.179310666
MsIH	1025.62	4.736303584	1091.186667	8.096249835	2613.616667	4.920767836
	Conc. (μg/mL)	Conc. RSD	Conc. (μg/mL)	Conc. RSD	Conc. (μg/mL)	Conc. RSD
Conc. in treated MsIH (ng/mL)	3.895223237	5.020750559	0.683407954	15.25934	0.345703112	9.888498068
Conc. in original MsIH (μM)	0.024		0.003		0.002	

	56 -> 56 Fe [H2]		55 Mn [He]		55 -> 55 Mn [H2]	
Sample Name	CPS	CPS RSD	CPS	CPS RSD	CPS	CPS RSD
standard 0ppb	2252.443333	6.185253323	41.11	16.88532236	361.13	9.292229275
standard 0.5ppb	9149.233333	1.755206131	874.5	7.276315051	10697.84333	2.442297211
standard 1ppb	15211.09667	1.289113597	1744.593333	5.924474673	21341.76	1.383506717
standard 2ppb	28535.75667	0.207225371	3372.653333	4.256923314	42395.55333	0.605860747
standard 5ppb	67739.79333	0.764375938	8418.853333	1.050561728	105240.26	0.624681009
standard 10ppb	132273.9767	0.716547987	16786.95667	2.079189515	210633.9867	0.50810842
standard curve	y = 12997.78 * x + 2450.57		y = 1674.29 * x + 44.06		y = 21028.42 * x + 274.13	
HNO3 blank	1784.6	4.992564422	48.89333333	21.91680999	453.36	7.014462868
H2O blank	895.6066667	3.801444784	28.88666667	52.03423437	232.2333333	2.987319823
MslH	6894.836667	2.252060428	7089.366667	1.176365058	90862.70333	0.627115713
	Conc. (μg/mL)	Conc. RSD	Conc. (μg/mL)	Conc. RSD	Conc. (μg/mL)	Conc. RSD
Conc. in treated MslH (ng/mL)	0.341924903	3.493849818	4.207944716	1.183721738	4.307911762	0.629013439
Conc. in original MslH (μM)	0.002		0.019		0.020	

	59 -> 59 Co [H2]		59 -> 75 Co [O2]		60 -> 60 Ni [H2]	
Sample Name	CPS	CPS RSD	CPS	CPS RSD	CPS	CPS RSD
standard 0ppb	18.89	36.74725263	8.89	43.25256235	38.89333333	64.33586047
standard 0.5ppb	2957.006667	0.627827576	1164.53	7.955951313	295.57	3.445254262
standard 1ppb	5886.693333	3.622743838	2246.886667	4.079530411	465.5766667	15.70927783
standard 2ppb	11661.81	2.047501849	4454.066667	2.186384871	998.9566667	5.23775412
standard 5ppb	29400.51333	1.16985414	11179.34	0.358595917	2363.573333	1.767217167
standard 10ppb	58714.30667	0.698658506	22264.15333	3.378221613	4629.636667	5.713026506
standard curve	y = 5871.78 * x + 1.87		y = 2224.87 * x + 26.31		y = 459.19 * x + 49.55	
HNO3 blank	80.00666667	19.09247436	22.22333333	31.21742494	50	33.34
H2O blank	40	22.04950396	8.89	78.0827449	52.22666667	9.750028375
MslH	75.55666667	25.08844402	27.78	6.920721369	1083.41	2.682809556
	Conc. (μg/mL)	Conc. RSD	Conc. (μg/mL)	Conc. RSD	Conc. (μg/mL)	Conc. RSD
Conc. in treated MslH (ng/mL)	0.012549404	25.72487932	0.000662375	130.4595246	2.251517464	2.81137778
Conc. in original MslH (mM)	0.000		0.000		0.010	

	60 Ni [He]		60 -> 76 Ni [O2]		62 -> 62 Ni [H2]	
Sample Name	CPS	CPS RSD	CPS	CPS RSD	CPS	CPS RSD
standard 0ppb	142.23	9.469122044	14.44333333	35.25581458	10.00333333	57.71578832
standard 0.5ppb	1532.346667	1.274326187	77.78	23.60527444	53.33666667	45.06830801
standard 1ppb	3128.16	3.263279967	211.12	24.47747485	75.56	37.52157432
standard 2ppb	5884.473333	4.027739678	320.0133333	4.773317461	151.1166667	33.40333829
standard 5ppb	14412.69667	1.425146877	823.3833333	6.073112979	412.24	6.122832968
standard 10ppb	28297.85333	0.750317755	1536.79	3.075488456	730.04	9.825262432
standard curve	y = 2814.74 * x + 220.85		y = 152.66 * x + 26.57		y = 73.077 * x + 13.40	
HNO3 blank	155.56	7.524133359	8.89	94.3785185	11.11	96.43371298
H2O blank	115.56	1.663704047	20	50	6.666666667	132.325366
MslH	6556.956667	1.711474812	332.24	5.526180611	196.6766667	12.22205645
	Conc. (μg/mL)	Conc. RSD	Conc. (μg/mL)	Conc. RSD	Conc. (μg/mL)	Conc. RSD
Conc. in treated MslH (ng/mL)	2.251045978	1.77113082	2.00236816	6.006498047	2.508053915	13.11541764
Conc. in original MslH (μM)	0.010		0.009		0.011	

	63 Cu [He]		63 -> 63 Cu [H2]		65 -> 65 Cu [H2]	
Sample Name	CPS	CPS RSD	CPS	CPS RSD	CPS	CPS RSD
standard 0ppb	230.01	12.63277677	113.3366667	32.75349712	47.78	10.66655332
standard 0.5ppb	4216.193333	2.750928576	415.5766667	6.915867411	161.1166667	9.32900792
standard 1ppb	8332.16	1.384950635	763.3766667	4.621467425	370.0166667	12.77189935
standard 2ppb	16325.52333	1.780286336	1432.333333	2.965343868	664.48	11.46520039
standard 5ppb	40032.74667	1.179083016	3334.87	7.615699773	1559.013333	9.762404807
standard 10ppb	79082.30333	0.971359989	6413.576667	1.070898657	2855.89	5.371992392
standard curve	y = 7882.57 * x + 398.57		y = 630.90 * x + 133.56		y = 282.58 * x + 71.77	
HNO3 blank	490.0266667	7.665890347	113.34	23.34349136	44.45	4.325256235
H2O blank	85.56	11.90175085	74.44666667	22.53407084	24.44333333	31.48539106
MsIH	2818.1	2.549446576	361.1266667	5.639950952	165.5633333	22.89789533
	Conc. (μg/mL)	Conc. RSD	Conc. (μg/mL)	Conc. RSD	Conc. (μg/mL)	Conc. RSD
Conc. in treated MsIH (ng/mL)	0.306946819	2.969418709	0.360696305	8.950163901	0.296787381	6.027349739
Conc. in original MsIH (μM)	0.001		0.001		0.001	

	64 -> 64 Zn [H2]		64 Zn [He]		95 Mo [He]	
Sample Name	CPS	CPS RSD	CPS	CPS RSD	CPS	CPS RSD
standard 0ppb	1328.983333	1.381748606	388.9066667	14.50458165	4.443333333	43.39872241
standard 0.5ppb	3437.123333	2.317819666	1111.183333	6.902463823	1875.726667	3.364718538
standard 1ppb	5623.266667	1.741380406	1913.51	4.018615688	3573.823333	2.525507168
standard 2ppb	10437.74	1.457732761	3379.333333	2.41110536	7161.693333	2.731449176
standard 5ppb	23730.39333	1.815571286	7720.783333	3.102921352	18049.55333	1.450363605
standard 10ppb	46265.11667	0.164842906	15305.69667	2.674358049	35247.26667	1.162177491
standard curve	y = 4500.64 * x + 1260.14		y = 1488.16 * x + 381.42		y = 3528.63 * x + 105.47	
HNO3 blank	666.7033333	2.291161834	242.2333333	6.92549798	156.6766667	22.21393624
H2O blank	41.11	4.676663577	22.22	8.65245903	37.77666667	28.36634333
MsIH	10253.18667	1.321366684	3365.996667	5.209912676	38.89	64.9029741
	Conc. (μg/mL)	Conc. RSD	Conc. (μg/mL)	Conc. RSD	Conc. (μg/mL)	Conc. RSD
Conc. in treated MsIH (ng/mL)	1.99817163	1.506522396	2.005553204	5.875723068	-0.018868129	N/A
Conc. in original MsIH (mM)	0.008		0.008		0.000	

	95 -> 95 Mo [H2]		95 -> 127 Mo [O2]	
Sample Name	CPS	CPS RSD	CPS	CPS RSD
standard 0ppb	1.11	173.2050808	4.443333333	114.6012337
standard 0.5ppb	393.3533333	5.872527906	2782.556667	5.169229542
standard 1ppb	925.6166667	9.544679952	5503.29	1.378373967
standard 2ppb	1730.156667	1.073381886	11235.09667	0.152264917
standard 5ppb	4364.03	5.161757594	27918.43333	0.510383794
standard 10ppb	8930.306667	1.447368934	55318.77333	0.46746894
standard curve	y = 892.11 * x - 26.58		y = 5536.16 * x + 57.26	
HNO3 blank	46.66666667	7.146431249	132.23	29.22107163
H2O blank	12.22333333	41.65005276	45.55666667	11.1775404
MsIH	6.666666667	173.2050808	26.67	54.48946306
	Conc. (μg/mL)	Conc. RSD	Conc. (μg/mL)	Conc. RSD
Conc. in treated MsIH (ng/mL)	0.037271923	34.72706099	-0.005525522	N/A
Conc. in original MsIH (μM)	0.000		0.000	

Supplementary Table 4. Analysis of *B*-factors for metals in the MslH:MslAΔTrp21 structure. [a]

	H_2O	Mg	Ca	Mn	Fe
metal	(18.80)	30.35	**43.35**	54.85	57.16
Oε Glu44	42.13	43.01	47.84	45.89	46.52
Oδ Asn87	40.20	40.22	42.49	40.78	40.72
Nε His259	35.17	36.30	34.21	36.69	36.33
Nε His261	33.55	34.76	36.07	35.64	35.74
w1	40.08	41.68	38.62	42.97	42.82
w2	40.08	41.38	41.27	41.68	43.23
average [b]	38.54	39.56	40.08	40.61	40.89

[a)]Each atom for the *B*-factor calculation corresponds with that in Supplementary Figure 14. [b)]Average was calculated without the *B*-factor value of the metal.

Supplementary Table 5. Oligonucleotides used for construction of mutant enzymes.

Mutant name	Oligonucleotide sequence
**MslH** [a]	
mslH-F	5’-ATAGTAACATATGACCCGGCTGACGGTGGCCCTGTC-3’
mslH-R	5’-CGTCCCATTCGCCAATCCGGATATAGTTCC-3’
Asp11A-F	5’-GCGGCAGCCATATGACCCGGCTGACGGTGGCCCTGTCCGGC**GCC**TGCATGGTGACGCGAGGAGGACTG-3’
Asp11A-R	Same as MslH-R
Glu44A-R	5’-CTGGGGACGAC**CGC**CAGATTGGTGACGGCGAAGTCG-3’
Glu44A-F	5’-CAATCTG**GCG**GTCGTCCCCAGCGACGGACGCGGA-3’
Asn87A-R	5’-GAGCACGCCCTCCGTTCCCAGGTCCATGGCGTG**GGC**GTTGGCGCAGCCCAGTACGGAGAATC-3’
Asn87A-F	5’-CTGGGAACGGAGGGCGTGCTC-3’
His88A-R	5’-GAGCACGCCCTCCGTTCCCAGGTCCATGGC**GGC**GTTGTTGGCGCAGCCCAGTACGGAGAATC-3’
His88A-F	Same as Asn87A-F
Asp91A-R	5’-GAGCACGCCCTCCGTTCCCAG**GGC**CATGGCGTGGTTGTTGGCGCAGCCCAGTACGGAGAATC-3’
Asp91A-F	Same as Asn87A-F
His259A-R	5’-CACGCTCACCACGACGAGGTCC-3’
His259A-F	5’-GGACCTCGTCGTGGTGAGCGTG**GCC**TCCCACGAGCCCGGCCCGACGCCCGA-3’
His261A-R	Same as His259A-R
His261A-F	5’-GGACCTCGTCGTGGTGAGCGTGCACTCC**GCC**GAGCCCGGCCCGACGCCCGA-3’
His295N-R	5’-TCCGCGCAGGAA**GTT**CGGCCCGTGTCCGACCACGG-3’
His295N-F	5’-ACGGGCCG**AAC**TTCCTGCGCGGAGTGGAGCTGTA-3’
**MslA** [b]	
Cys13/19S-F	5’-AACGACTTCGCCGGC**AGC**GGCTACGCGATCGTC**AGC**TTCTGGTGA-3’
Cys13/19S-R	5’-GCCGGCGAAGTCGTTGCAGCTTCCGACGCCGAGGCACTTCGTGAG-3’
mslA-F	5′-GAATGGTGCATGCAAGGAGATGGCG-3′
mslA-ΔTrp21-R	5′-ACAGAAGCTTTCAGAAGCAGACGATCGCG-3′
mslA-Trp21G-R	5′-ACAGAAGCTTTCACCCGAAGCAGACGATCGCG-3′

F, forward primer; R, reverse primer. Mutated codons and overlapping sequences between each primer set are underlined and bolded, respectively. [a)] pET28a vector encoding the *mslH* gene was used as the template. [b)] pET21 vector encoding the *mslA* gene was used as the template.

Supplementary Table 6. Reagents used.

Reagents	Vender	Catalog number
Potassium Dihydrogen Phosphate	wako	169-04245
Sodium Chloride	wako	195-01663
Ammonium Chloride	wako	017-02995
Disodium Hydrogenphosphate	wako	197-02865
Magnesium Sulfate Heptahydrate	wako	131-00405
D(+)-Glucose	wako	049-31165
Glycerol	wako	075-00611
Calcium Chloride Dihydrate	wako	031-25031
Thiamine Hydrochloride	wako	209-00853
Thymidine	sigma	T9250-1G
Adenosine	wako	011-24593
Guanosine	sigma	G6752-1G
Cytidine	sigma	C4654-1G
Sodium Dihydrogenphosphate	wako	197-09705
Imidazole	wako	095-00015
2-Amino-2-hydroxymethyl-1,3-propanediol	wako	207-06275
(+/-)-Dithiothreitol	wako	049-08972
Gel Filtration Calibration Kits	cytiva	28403842
L-FDLA	TCI	A5523
Deuterium Oxide (99.8 %)	wako	049-34242
2.0 M Magnesium chloride hexahydrate	Hampton Research	HR2-559
1.0 M Tris hydrochloride pH 8.0	Hampton Research	HR2-937-11
100% Polyethylene glycol 400	Hampton Research	HR2-603
